# miR-17-5p/*HOXA7* Is a Potential Driver for Brain Metastasis of Lung Adenocarcinoma Related to Ferroptosis Revealed by Bioinformatic Analysis

**DOI:** 10.3389/fneur.2022.878947

**Published:** 2022-05-25

**Authors:** Quanfang Chen, Qingyun Pan, Han Gao, Yingju Wang, Xiaoning Zhong

**Affiliations:** Department of Respiratory Medicine, First Affiliated Hospital of Guangxi Medical University, Nanning, China

**Keywords:** *HCP5*, hsa-miR-17-5p, *HOXA7*, lung adenocarcinoma, ferroptosis, neurovascular, brain metastasis, biomarker

## Abstract

**Objectives:**

Present study aims to identify the essential mRNAs responsible for the development of brain neurovascular-related metastases (BNM) among lung adenocarcinoma (LUAD) patients. Further, we attempted to predict brain metastases more accurately and prevent their development in LUAD patients.

**Methods:**

Transcriptome data analysis was used to identify differentially expressed mRNAs (DEMs) associated with brain metastasis, and thereby the ferroptosis index (FPI) is calculated using a computational model. Meanwhile, the DEmRNAs linked with FPI, and brain metastasis were derived by the intersection of these two groups of DEMs. We also constructed a ceRNA network containing these DEmRNAs, identifying the *HCP5* /hsa-miR-17-5p/*HOXA7* axis for analysis. Further, a clinical cohort was employed to validate the regulatory roles of molecules involved in the ceRNA regulatory axis.

**Results:**

Here we report the development of a ceRNA network based on BNM-associated DEMs and FPI-associated DEmRNAs which includes three core miRNAs (hsa-miR-338-3p, hsa-miR-429, and hsa-miR-17-5p), three mRNAs (*HOXA7, TBX5*, and *TCF21*), and five lncRNAs (*HCP5, LINC00460, TP53TG1*). Using gene set enrichment analysis (GSEA) and survival analysis, the potential axis of *HCP5* /hsa-miR-17-5p/*HOXA7* was further investigated. It is found that *HOXA7* and ferroptosis index are positively correlated while inhibiting tumor brain metastasis. It may be that *HCP5* binds competitively with miR-17-5p and upregulates *HOXA7* to increase iron death limiting brain cancer metastases

**Conclusions:**

The expression of both *HOXA7* and *HCP5* is positively correlated with FPI, indicating a possible link between ferroptosis and BNM. According to the results of our study, the ferroptosis-related ceRNA *HCP5* /hsa-miR-17-5p/*HOXA7* axis may contribute to the development of BNM in LUAD patients.

## Introduction

Lung adenocarcinoma (LUAD) is the most common histological subtype of non-small cell lung cancer (NSCLC) which frequently occurs in peripheral lung tissue while accounting for approximately 70% of NSCLC and 40% of lung tumors (1–4). There has been an increase in LUAD morbidity and mortality rates worldwide in recent years (5–7). LUAD patients have an extremely low average survival rate (8, 9). In some studies, it has been estimated that there are 90,000 LAUD cases and around 8,00,000 LUAD deaths worldwide each year (10, 11). The 5-year survival rate for LUAD is <20% even though treatment options have evolved in recent times (12, 13). Such a low survival rate for LUAD patients may be attributed to its high infiltration and metastasis rates (14). Metastasis of LUAD often occurs in the brain, where the incidence and prognosis are poor, which poses a significant risk to the patients' health (15, 16). This suggests that brain metastases are a significant cause of treatment failure and death in patients with LUAD. Hence, to improve LUAD patients' prognosis, it is critical to identify biomarkers associated with brain metastases (17).

It has been demonstrated that ferroptosis plays a significant regulatory role in the treatment of many diseases, including cardiovascular, kidney, and oncological disorders (18–21). A ferroptosis process has been observed to influence tumor growth in oncological diseases and its role is expected to be exploited as an anti-cancer therapy target (22, 23). Ferroptosis is associated with the development of NSCLC and inducing ferroptosis may improve the therapeutic potential of NSCLC (24, 25). Some studies suggest that ferroptosis in lung cancer is regulated by USP35, which is thought to affect the growth and progression of the disease and may represent a new therapeutic target (22). Furthermore, ferroptosis appears to enhance the antitumor effects of conventional chemotherapy and radiotherapy (26–28). However, an exact mechanism of action for ferroptosis in LUAD brain metastases is still unknown.

Additionally, the ceRNA network is involved in regulating multiple tumor types (29–32). Several studies have shown that the ceRNA network has an association with lung cancer prognosis and NSCLC cell proliferation (33, 34). There was previously a finding that lincRNA00494 as ceRNA suppresses the proliferation of non-small cell lung cancer cells by regulating SRCIN1 expression (33). Studies have also shown that ceRNA regulatory networks can be used to construct models for assessing the prognosis of patients with malignancies (35). Thus, ferroptosis-related ceRNA may be a good candidate for the identification of new biomarkers in lung cancer diagnosis and prognosis (36).

The major focus of our study was to identify the major mRNAs involved in the development of BNM among LUAD patients and to explore the potential of the ceRNA regulation network for identifying new biomarkers. In addition, this research also intended to uncover any possible therapeutic target to monitor and improve the risk of BNM in LUAD patients with the possibility to extend their survival period.

## Methods

### Data Download

Due to the lack of BNM information in The Cancer Genome Atlas (TCGA, accessible at https://portal.gdc.cancer.gov/) project, we downloaded the transcriptome profile of LUAD brain neurovascular-related metastases (BNMs) from GSE141685 in the Gene Expression Omnibus (GEO) database (37). This BNM-LUAD dataset is containing bulky RNA sequencing (RNA-seq) data from surgically resected brain metastases of 6 LUAD patients. To investigate the potential molecules and pathways involved in LUAD brain metastasis, we have compared the BNM-LUAD bulk RNA-seq data with nM-LUAD (LUAD with no metastasis) bulk RNA-seq data of 384 patients from the TCGA database. From the TCGA database, we also downloaded miRNA sequences and clinical characterization data on nM-LUAD. In addition, the raw transcript RNA-seq data from the BNM-LUAD dataset were stored in Sequence Read Archive (SRA) file format and downloaded locally for subsequent RNA-seq analysis.

### Data Preparation and Recruitment of Patients

The Kallisto software was used to quantify transcript abundance on bulk RNA-seq data (38). The transcripts per million (TPM) units of gene expression are included in the quantitative results of the transcriptome data and the log (TPM+1) for gene expression is calculated. In the next step, we applied the “ComBat” function from the limma package to remove the batch effect and pooling of data (39). To validate the findings of this study, an independent cohort of LUAD patients was also utilized. The Institutional Ethics Review Board of The First Affiliated Hospital of Guangxi Medical University has approved this study and informed written consent was also obtained from all the patients. We randomly collected and then examined 29 tumor tissue samples from the First Affiliated Hospital of Guangxi Medical University where this cohort was enrolled as a clinical cohort. A combination of diagnostic imaging and histology is used to better understand the clinical diagnosis. To obtain tumor tissue samples, surgical excision was used, and excess tumor tissues were collected only after a confirmed pathological diagnosis.

### Identification of FPI-Related Genes

In the TCGA nM-LUAD dataset, we used the GSVA package to calculate the enrichment scores of ferroptosis-related gene sets (including *ACSL4, ALOX15, COQ10A, COQ10B, FDFT1, GPX4, HMGCR, SLC3A2, SLC7A11, NFE2L2, NOX1, NOX3, NOX4, and NOX5*). This followed the ferroptosis index (FPI) calculation by applying the standardized method as described previously (40, 41). These FPI scores are used to quantify the likelihood of ferroptosis of cells within a tumor or tissue and based on median FPI scores, nM-LUAD patients are divided into high and low FPI groups. We next performed differential expression analyses for lncRNAs, miRNAs, and mRNAs between the low and high FPI groups with the limma package (*p* < 0.05). These differentially expressed mRNAs (DEMs) were defined as ferroptosis-related or FPI-related mRNAs (including FPI-related DEmRNAs, DEmiRNAs, and DElncRNAs). As described previously by utilizing the clusterProfiler R package the FPI-associated DEmRNAs are used for functional pathway analysis including the Kyoto Encyclopedia of Genes and Genomes (KEGG) and gene ontology (GO) (42–44).

### Identification of Brian Metastasis-Related Genes

In this study, a significance level of *p* < 0.05 was set as the cut-off criterion. We have also performed a differential expression analysis between the BNM and no metastasis groups using the “limma” package. The calculated DEMs were named BNM-related mRNAs which are associated with the development of BNM in LUAD.

### Identification of Genes Potentially Regulated by Identified FPI-Related miRNAs

We performed intersection analysis studies while including FPI-related DEmRNAs and BNM-related DEmRNAs that may play a crucial role in ferroptosis and brain metastases. Firstly, the miRcode database confirmed a relationship between delncRNAs and demiRNAs (45, 46). After that, we used miRWalk (http://mirwalk.umm.uni-heidelberg.de/) to identify shared DEmiRNAs and DEmRNAs between FPI and BNM (47). As a result, ceRNA networks were constructed based on DElncRNA-DEmiRNA interaction pairs and DEmiRNA-DEmiRNA interaction pairs.

### Identification of Genes as the Target of FPI-Related miRNAs Associates to FPI, Brain Metastasis, and FPI-Related miRNAs

The correlation analysis was used to screen possible lncRNA-miRNA and miRNA-mRNA interaction pairs further. A Cox regression analysis was conducted on the molecules in these interaction pairs to predict the probability of survival of LUAD patients after 1, 3, and 6 years. To quantify and predict the survival rates of LUAD patients, the time-dependent receiver operating characteristic curve (ROC) and area under the curve (AUC) were used. We used the “survival” R package to analyze survival data and the “survminer” R package to visualize the data. A Pearson's method and linear regression analysis were then performed to determine the correlation between the variables. The “ggplot2” package was used to plot the heat map and scatter plots of the statistical tests.

### Gene Set Enrichment Analysis

GSEA (Gene Set Enrichment Analysis) is a bioinformatics technique for exploring a specific set of functional genes (48, 49). Two groups are generated depending on the expression level of the target gene and critical pathway analysis was then conducted using GSEA. We used the reference dataset “c2.cp.v7.2.symbols.gmt [Curated]” for the enrichment analysis. All adjusted *p-*values < 0.05 were considered significantly enriched.

### Quantitative Reverse Transcription PCR (qRT-PCR)

To quantify the abundance of mRNAs and miRNAs in samples, the total RNA was extracted from cells using Trizol reagent (Invitrogen) and quantified using Nanodrop (Thermo Scientific, Waltham, USA). The expression of miRNAs was determined by stem-loop qPCR (Taq-Man) as mentioned previously as a reference. We conducted a qPCR on the cDNA template with TB GreenTM Premix Ex TaqTM II (Takara; RR820A) to determine the levels of mRNA. In addition, qPCR primer sets for miRNAs as well as mRNAs were obtained from RiboBio while mRNA qPCR primers have been synthesized by Sangon (Shanghai, China), as described in previous studies (50). For the relative quantification of gene expression levels, the CT method was performed in triplicates while GAPDH or U6 are included as internal controls.

### Statistical Methods

Statistical analyses were performed using R software (version 4.0.2). The Kaplan-Meier method was used, and survival curves were plotted for survival analyses. Unless otherwise stated, *p* < 0.05 was found to be significant.

## Results

### DEGs Related to Ferroptosis Index in LUAD

**Figure 1:**
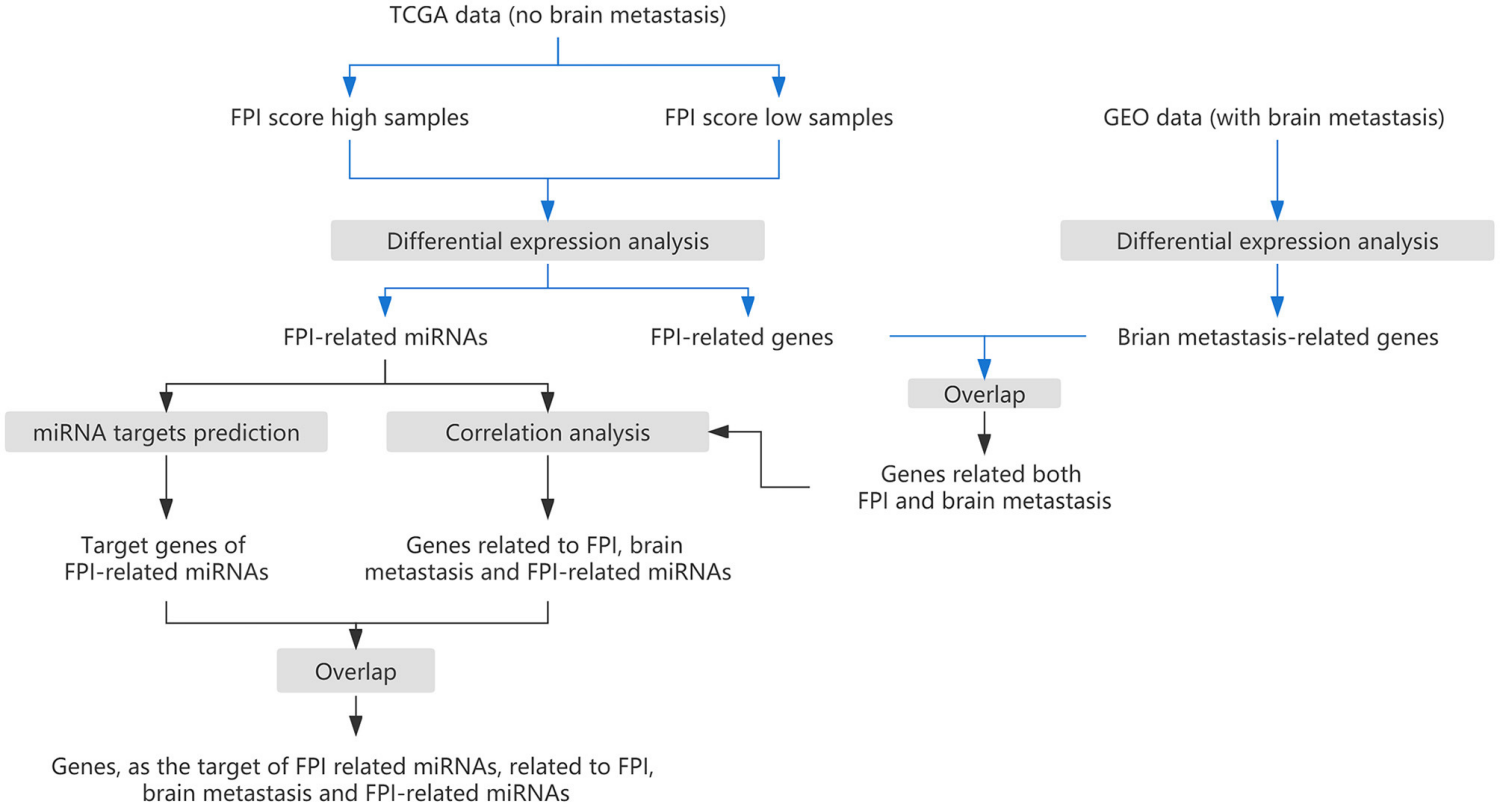
The research strategy for this study.

**Figure 2:**
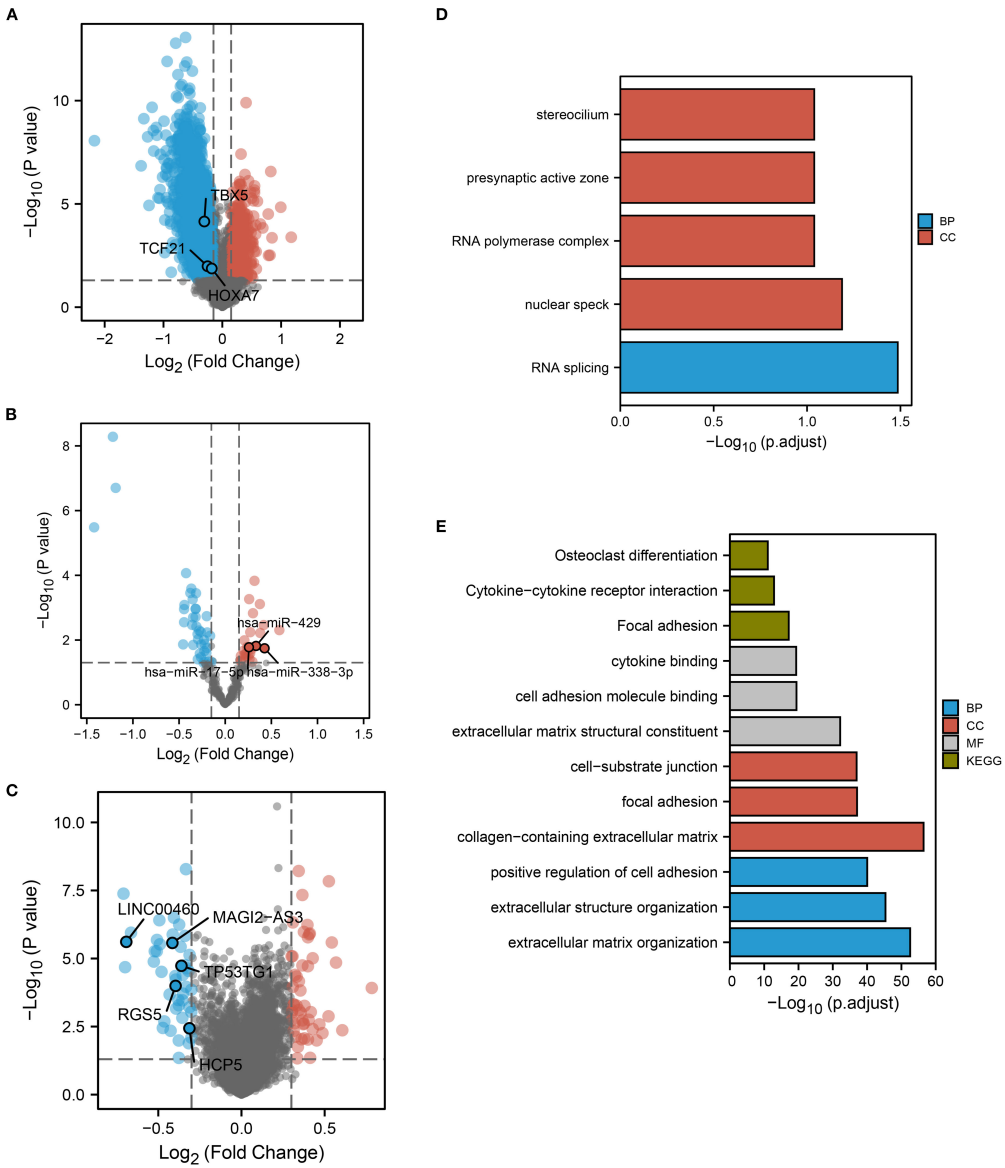
FPI-related DEGs and functional pathway analysis. Volcano plots showed FPI-related **(A)** DEmRNAs, **(B)** DEmiRNAs, and **(C)** DElncRNAs. The plots show the predicted RNAs in ceRNA networks. Red dots indicate upregulated genes in low FPI groups. The low FPI fraction group **(D)** upregulated **(E)** downregulated GO and KEGG pathways.

The flow chart of this study is shown in Figure 1. We calculated the probability of ferroptosis based on the core mRNAs associated with ferroptosis and it was termed the ferroptosis index (FPI) (40). According to their median FPI values, 384 LUAD patients without metastasis (nM-LUAD) in the TCGA-LUAD project were classified into high or low FPI groups. We then identified 5,958 DEmRNAs, 2,380 lncRNAs, and 68 DEmiRNAs to analyze the differences between the low and high FPI groups (Figures 2A–C). It has been proposed that these dysregulated mRNAs are associated with ferroptosis and are known as FPI-related mRNAs. The DEmRNAs that were upregulated in the low FPI group were enriched in RNA splicing, stereocilium, presynaptic active zone, and RNA polymerase complex (Figure 2D). Conversely, pathways that were downregulated in the low FPI group included collagen–containing extracellular matrix, positive regulators of cell adhesion, focal adhesion, and cell adhesion molecule binding (Figure 2E). The analysis described above can identify the mRNAs associated with FPI in LUAD, as well as their possible functions.

### Identification of Shared DEmRNAs and Construction of ceRNA Network

**Figure 3:**
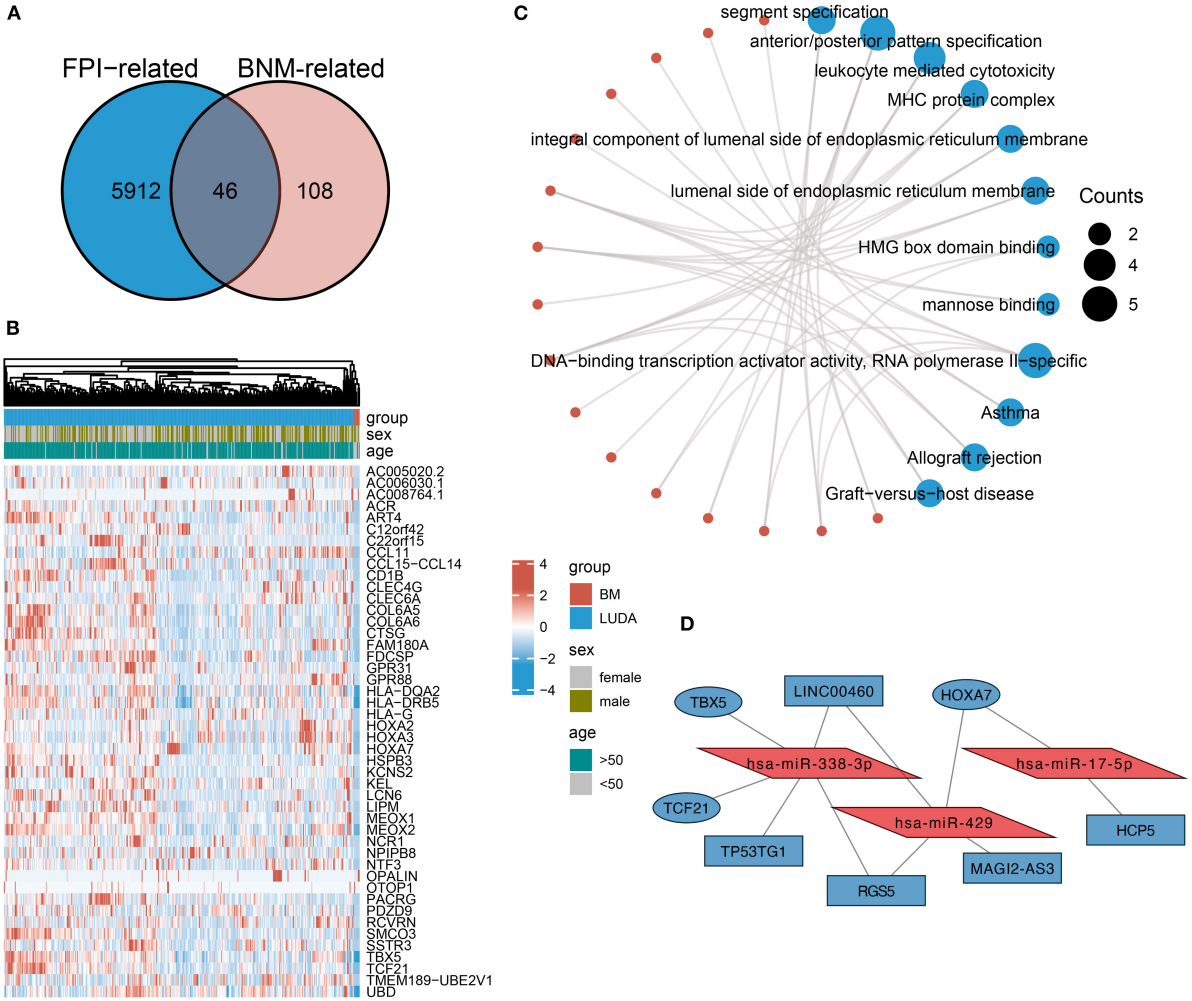
An intersection analysis of FPI- and BM-associated DEmRNAs, and the construction of ceRNA networks. **(A)** Venn plot showed 46 shared DEmRNAs among FPI- and BNM-associated DEmRNAs. In BNM and the low FPI group, 44 of these shared DEmRNAs were downregulated. **(B)** Expression of genes shared by brain metastases and non-metastases. **(C)** The functional pathways of the shared DEmRNAs downregulated in BNM. **(D)** Construction of ceRNA networks based on the shared DEmRNAs.

To identify DEmRNAs shared with both FPI and BNM, the differential analysis between BNM-LUAD and nM-LUAD revealed 154 BNM-related DEmRNAs. Then, the intersection analysis of BNM-related DEmRNAs and FPI-related DEmRNAs identified 46 downregulated 44 mRNAs that are common to both brain metastases and ferroptosis (Figures 3A,B). BNM-LUAD downregulated 44 out of the 46 shared mRNAs. Downregulated mRNAs were mostly found to be enriched in leukocyte-mediated cytotoxicity MHC protein complexes, segment specification, HMG box domain binding, allograft rejection, and graft-vs.-host diseases (Figure 3C). Immunologically-related pathways may likely be downregulated in patients with LUAD, contributing to brain metastases.

Based on these 44 DEmRNAs [RT1], we constructed a ceRNA network (Figure 3D). The DElncRNAs and DEmiRNAs in the network were derived from DEMs related to FPI. The ceRNA network consists of three core miRNAs (hsa-miR-338-3p, hsa-miR-429, and hsa-miR-17-5p), three mRNAs (*HOXA7, TBX5*, and *TCF21*), and five lncRNAs (*HCP5, LINC00460, TP53TG1*, RGS5, and MAGI2-AS3).

### Identification of Potential *HCP5*/hsa-miR-17-5p/*HOXA7* Pathways From the ceRNA Network

**Figure 4:**
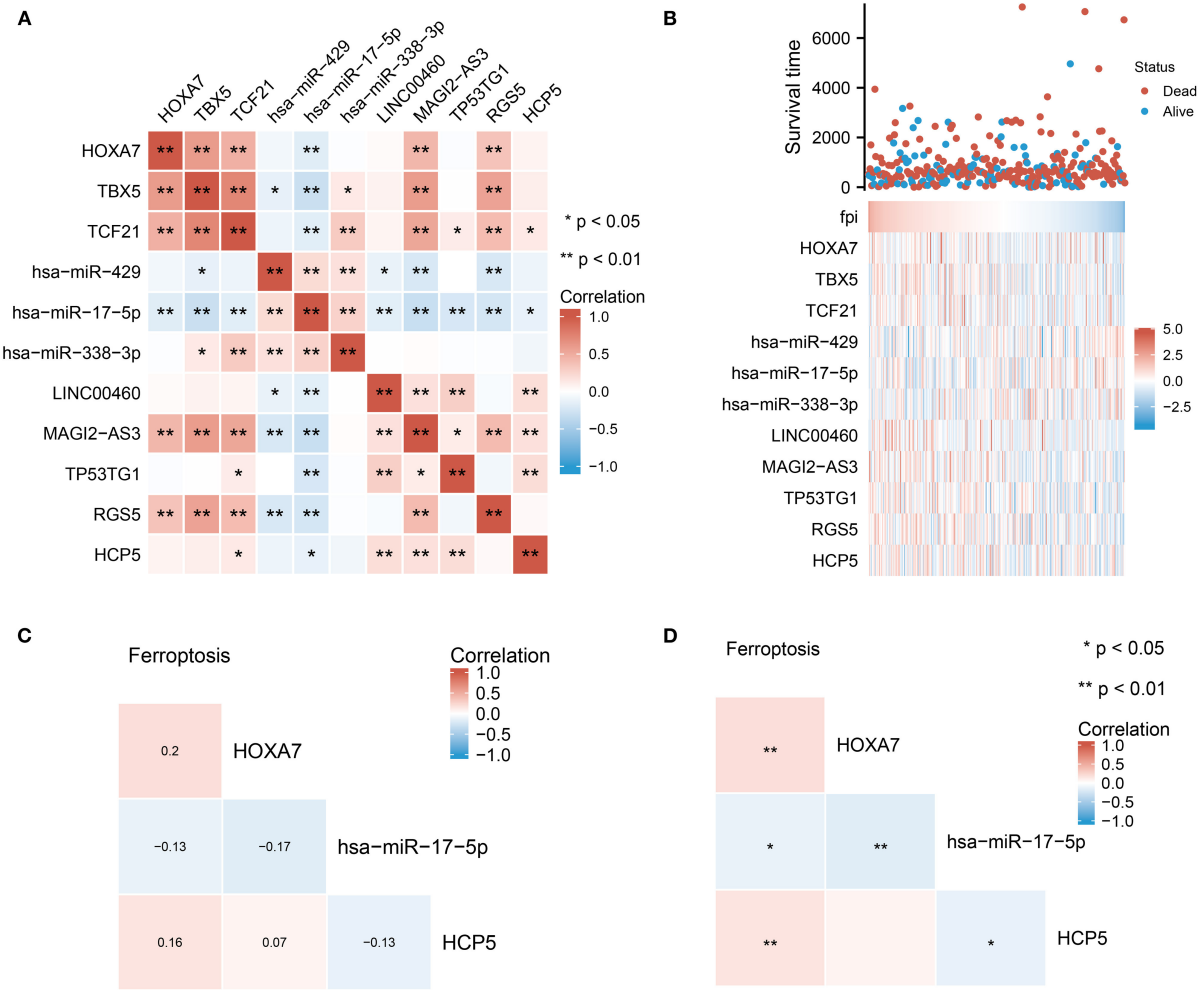
Identification of potential *HCP5* /hsa-miR-17-5p/*HOXA7* pathways from the ceRNA network. **(A)** Correlation analysis of each element in the ceRNA network. **(B)** FPI and the expression of individual elements in the *HCP5* /hsa-miR-17-5p/*HOXA7* pathway have statistically significant connections. **(C,D)** Ferroptosis is significantly linked to each molecule in the *HCP5* /hsa-miR-17-5p/*HOXA7* pathway; **(C)** shows the correlation coefficients and **(D)** shows the statistical differences.

Based on the constructed ceRNA network, we performed correlation analysis to identify potential pathways. An analysis of the correlation between these 11 dysregulated molecules is depicted in Figure 4A. Significant correlations (*p* < 0.01) were observed between the mRNA and miRNA groups. The heat map in Figure 4B shows the expression of these 11 dysregulated molecules in LUAD. The correlation studies identified the *HCP5* /hsa-miR-17-5p/*HOXA7* axes associated with ferroptosis and BNM from the ceRNA network (Figures 4C,D). There was a negative correlation between the expression of *HCP5* and hsa-miR-17-5p (Pearson r = −0.13, p 0.05). Also, the expression of hsa-miR-17-5p and *HOXA7* exhibited a negative correlation (Pearson r = −0.17, p 0.01). Additionally, we found a positive correlation between FPI and HXOA7 and *HCP5* (*p* < 0.01), as well as a negative correlation between FPI and hsa-miR-17-5p (*p* < 0.05). *HCP5* overexpression was also linked to a superior primary therapy outcome, suggesting that it may improve the prognosis of LUAD patients (Table 1). Thus, ceRNA *HCP5* /hsa-miR-17-5p/*HOXA7* may play a role in the development of LUAD.

**Table 1:** The relation between HCP5 expression levels and LUAD clinicopathological features.

**Characteristic**	**Low expression of**	**High expression of**	***p*-value**
	**HCP5 (*n* = 267)**	**HCP5 (*n* = 268)**	
**Age**, ***n*** **(%)**			0.187
< =65	135 (26.2%)	120 (23.3%)	
>65	122 (23.6%)	139 (26.9%)	
**Gender**, ***n*** **(%)**			0.022
Female	129 (24.1%)	157 (29.3%)	
Male	138 (25.8%)	111 (20.7%)	
**Smoker**, ***n*** **(%)**			0.139
No	31 (6%)	44 (8.4%)	
Yes	229 (44%)	217 (41.7%)	
**Race**, ***n*** **(%)**			0.371
Asian	2 (0.4%)	5 (1.1%)	
Black or African American	31 (6.6%)	24 (5.1%)	
White	204 (43.6%)	202 (43.2%)	
**T stage**, ***n*** **(%)**			0.172
T1	77 (14.5%)	98 (18.4%)	
T2	152 (28.6%)	137 (25.8%)	
T3	29 (5.5%)	20 (3.8%)	
T4	9 (1.7%)	10 (1.9%)	
**N stage**, ***n*** **(%)**			0.379
N0	176 (33.9%)	172 (33.1%)	
N1	43 (8.3%)	52 (10%)	
N2	40 (7.7%)	34 (6.6%)	
N3	0 (0%)	2 (0.4%)	
**M stage**, ***n*** **(%)**			0.621
M0	185 (47.9%)	176 (45.6%)	
M1	11 (2.8%)	14 (3.6%)	
**Pathologic stage**, ***n*** **(%)**			0.681
Stage I	144 (27.3%)	150 (28.5%)	
Stage II	62 (11.8%)	61 (11.6%)	
Stage III	46 (8.7%)	38 (7.2%)	
Stage IV	11 (2.1%)	15 (2.8%)	
**Primary therapy outcome**, ***n*** **(%)**			0.005
Progressive disease	45 (10.1%)	26 (5.8%)	
Stable disease	20 (4.5%)	17 (3.8%)	
Partial response	0 (0%)	6 (1.3%)	
Complete response	159 (35.7%)	173 (38.8%)	
**Residual tumor**, ***n*** **(%)**			0.172
R0	180 (48.4%)	175 (47%)	
R1	6 (1.6%)	7 (1.9%)	
R2	0 (0%)	4 (1.1%)	

### Correlation of *HCP5*, hsa-miR-17-5p, and *HOXA7* With LUAD Prognosis and FPI

**Figure 5:**
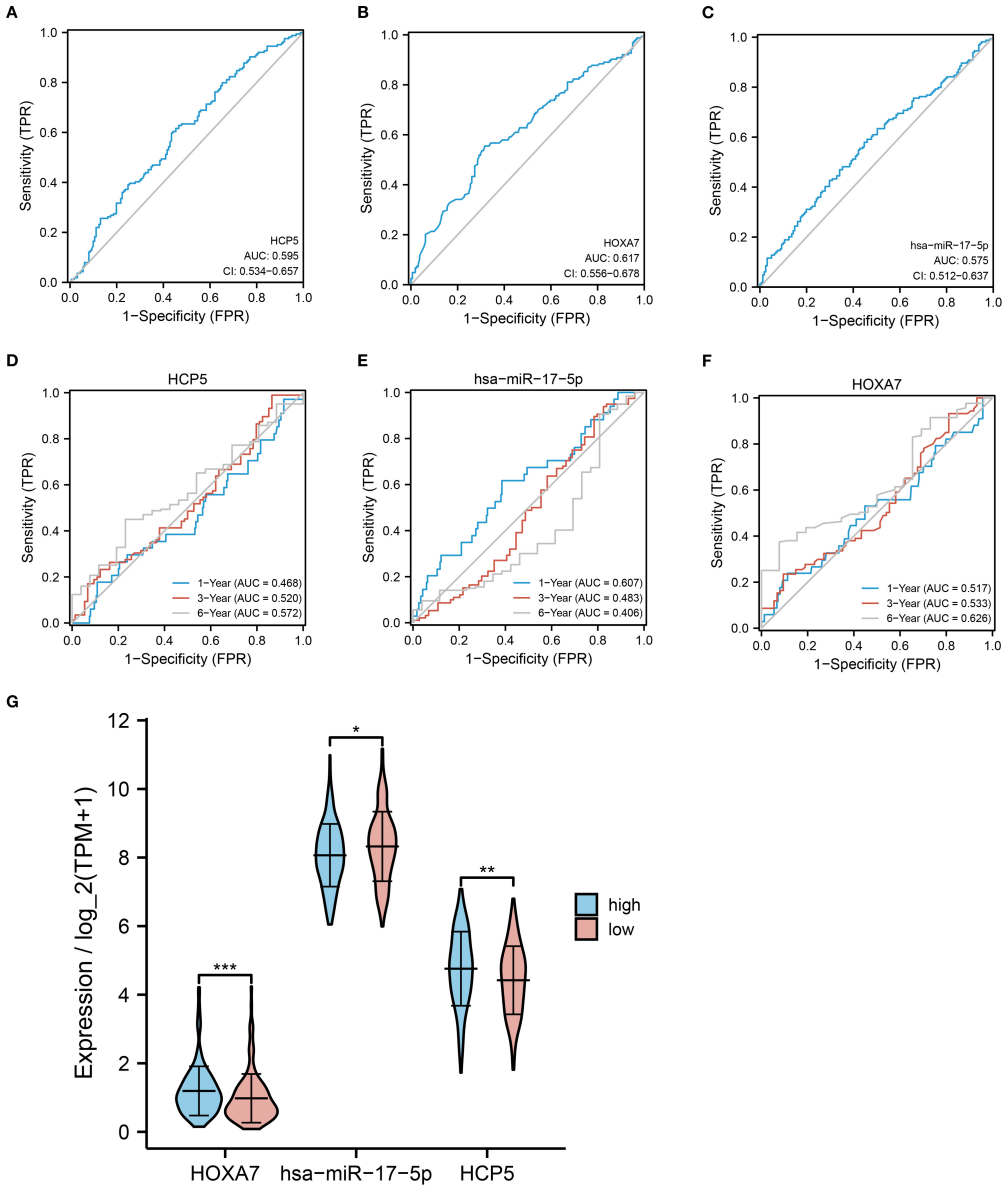
Correlation between *HCP5*, hsa-miR-17-5p, and *HOXA7* with LUAD prognosis and FPI. **(A–C)** ROC curves predicting high or low FPI scores for **(A)** *HCP5*, **(B)** *HOXA7*, **(C)** hsa-miR-17-5p. **(D–F)** ROC curves for predicting 1-, 3-, and 6-year survival rates. **(G)** Comparison of the expression of *HCP*, hsa-miR-17-5p, and *HOXA7* in high and low FPI groups. *P < 0.05, **P < 0.01, and ***P < 0.001.

To further evaluate the effect of *HCP5* /hsa-miR-17-5p/*HOXA7* in LUAD, ROC curves and analysis of differential expression were used to examine the relationship between *HCP5*, hsa-miR-17-5p, and *HOXA7* and LUAD prognosis and FPI. In ROC analysis, *HCP5* (AUC = 0.596, 95% CI: 0.534-0.657), *HOXA7* (AUC = 0.617, 95% CI: 0.556–0.678) and hsa-miR-17-5p (AUC = 0.575, 95% CI: 0.512–0.637) significantly predicted low FPI scores (Figures 5A–C). In addition, time-dependent ROC curves indicated that *HCP5*, HSA-miR-17-5p, and *HOXA7* might be associated with the survival of LUAD patients (Figures 5D,E). In addition, there was a statistically significant difference in the expression levels of *HCP5, HOXA7*, and hsa-miR-17-5p between the high and low FPI groups (Figure 5G). The potential *HCP5* /hsa-miR-17-5p/*HOXA7* axis is therefore thought to be related to ferroptosis.

### Clinical Correlation Analysis of *HCP5*, hsa-miR-17-5p, and *HOXA7*

**Figure 6:**
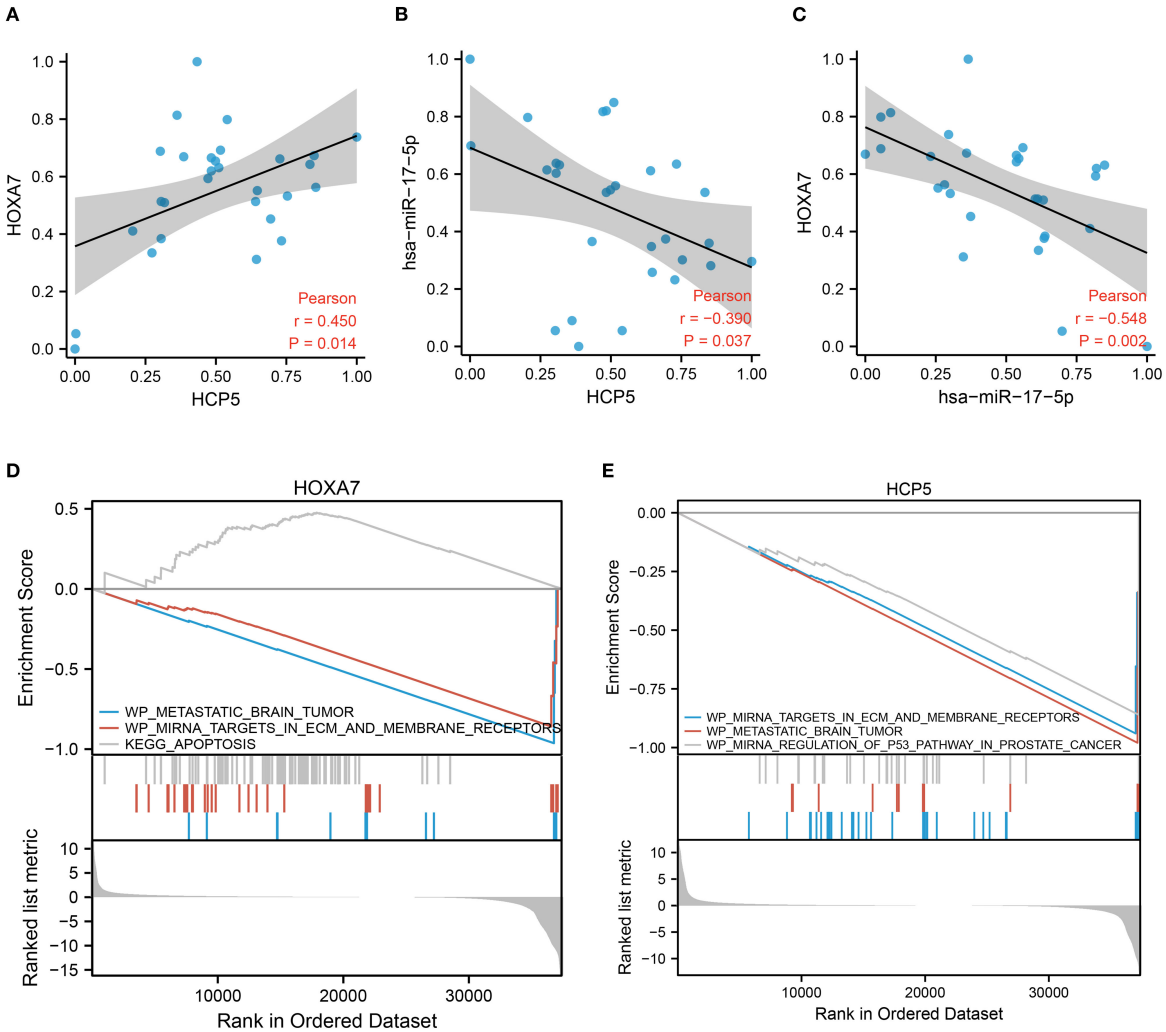
Validation of the *HCP5* /hsa-miR-17-5p/*HOXA7* pathway using GSEA and correlation analysis. **(A)** A positive correlation between the expression of *HOXA7* and *HCP5* in clinical cohort samples (Pearson *r* = 0.45, *p* = 0.014). **(B)** The expression of hsa-miR-17-5p and *HCP5* was negatively correlated (*r* = −0.39, *p* = 0.037). **(C)** The expression of *HOXA7* and hsa-miR-17-5p was negatively correlated (Pearson *r* = −0.548, *p* = 0.002). **(D,E)** GSEA demonstrates pathways involving *HOXA7* and *HCP5*.

To explain the potential effectiveness of the *HCP5* /hsa-miR-17-5p/*HOXA7* axis in LUAD at the clinical level, we performed qRT-PCR using available tumor samples from 29 LUAD patients. The expression of *HCP5* and *HOXA7* correlated positively in clinical samples (Figure 6A; Pearson r = 0.45, *p* = 0.014), while hsa-miR-17-5p correlated negatively with *HCP5* (Figure 6B; Pearson r = −0.39, *p* = 0.037) as well as *HOXA7* (Figure 6C; Pearson *r* = −0.548, *p* = 0.002). Furthermore, *HOXA7* expression and hsa-miR-17-5p expression were negatively related. In this study, lncRNA *HCP5* was found to act as an “RNA sponge” to sequester hsa-miR-17-5p, thereby decreasing the effects of hsa-miR-17-5p on the target *HOXA7* mRNA.

### Functional Analysis of *HCP5* and *HOXA7*

Using GSEA, we determined the relationship between pathway activation and inhibition when *HCP5* and *HOXA7* expression levels were high and low, respectively. The GSEA indicated that the tumor samples with elevated *HOXA7* expression downregulated miRNA targets in ECM and membrane receptors, as well as metastatic brain tumor pathways, while the apoptosis pathway was upregulated (Figure 6D). Additionally, the tumor samples with elevated *HCP5* expression downregulated miRNA targets in ECM and membrane receptors, metastatic brain tumors, as well as and miRNA regulation of the p53 pathway in prostate cancer (Figure 6E). Therefore, increased levels of *HOXA7* and *HCP5* were associated with the downregulation of tumor brain metastasis. Additionally, the expression of *HOXA7* and *HCP5* was positively correlated with FPI, indicating a strong relationship between ferroptosis and BNM.

## Discussion

According to this study, BNM and ferroptosis are linked in LUAD. It has been observed that the ferroptosis-related ceRNA *HCP5*/hsa-miR-17-5p/*HOXA7* axis may play an important role in the development of BNM in LUAD.

We examined the BNM-related DEMs in LUAD with and without metastatic tumors. In addition, we investigated DEmRNAs associated with FPI in LUAD patients without metastatic tumors between high and low FPI. Furthermore, the intersection studies identified DEmRNAs shared between BMogenesis and ferroptosis. Based on intersection analysis, a ceRNA network associated with BNM and ferroptosis was constructed. The *HCP5*/hsa-miR-17-5p/*HOXA7* axis is thought to play a central role in the ceRNA regulatory network of LUAD. In a clinical cohort, we validated the *HCP5* /hsa-miR-17-5p/*HOXA7* axis and confirmed our bioinformatic findings.

Previous studies suggest that *HOXA7* participates in the pathogenesis of LUAD, providing a new target for the early treatment of LUAD (51). In brain tumors, *HOXA7* is involved in glioma progression and affects patient prognosis (52). However, there is a lack of research on the role of *HOXA7* in the brain metastasis of lung cancer. Several studies have demonstrated that *HOXA7* is involved in tumor metastasis, suggesting that *HOXA7* expression is different in different types of cancer.

HOXA1 has diverse effects on tumor progression depending on the type of malignancy. By downregulating *HOXA7* and promoting cell migration and invasion, miR-196a plays a pro-cancer role in colorectal cancer (53). It has been shown that *HOXA7* expression is elevated in metastatic hepatocellular carcinoma, enhancing the metastasis of hepatocellular carcinoma, and is associated with a poor prognosis for patients (54). Also, *HOXA7* is upregulated in the ceRNA network in oral squamous cell carcinoma, increasing the invasion and migration of oral cancer cells (55). Our study is the first to investigate the role of *HOXA7* in brain metastasis in LUAD. In patients with brain metastases, *HOXA7* expression has been observed to be downregulated. Downregulation of *HOXA7* can lead to tumors becoming more likely to metastasize to the brain, which may be associated with a poor prognosis in patients who have brain metastases. Our findings showed a positive correlation between *HOXA7* and the ferroptosis index, suggesting *HOXA7* may promote ferroptosis. Some studies have revealed that negative regulation of ferroptosis can lead to increased resistance to chemotherapy in lung cancer patients with brain metastases (56). In addition, the promotion of ferroptosis inhibited brain metastasis in HER2-positive breast cancer (57). Thus, we hypothesized that *HOXA7* downregulation promotes LUAD brain metastasis, which may be related to ferroptosis induced by *HOXA7*.

Li et al. demonstrated that miR-17-5p is highly expressed in LUAD and is associated with patient prognosis as a novel marker for clinical diagnosis of NSCLC (58, 59). Moreover, miR-17-5p is a direct target of metastasis-associated LUAD transcript 1 (MALAT1) (60). Additionally, miR-17-5p has been shown to directly target MALT1 (metastasis-associated LUAD transcript 1). A study has shown that increased MALAT1 expression promotes brain metastasis in lung cancer and correlates with patient survival (61). Additionally, miR-17-5p is also involved in brain metastasis of other cancers (62). It is therefore thought that miR-17-5p promotes LUAD brain metastasis, and this may be due to the inhibition of ferroptosis. Even though no studies have indicated a link between miR-17-5p and ferroptosis, some studies have found that miR-17-5p can resist lung cancer cell apoptosis (63). In the present study, miR-17-5p has been observed to negatively regulate *HOXA7*, thus possibly promoting LUAD brain metastasis. According to our correlation analysis, miR-17-5p promotes LUAD brain metastasis by inhibiting ferroptosis and negatively targeting *HOXA7*.

*HCP5* affects tumor progression differently depending on the type of malignancies. Several studies have explored the relationship between *HCP5* and metastasis and prognosis in cancer (64). It was reported by Jiang et al. that *HCP5* was highly expressed in tumor tissues of LUAD patients and contributed to epithelial-mesenchymal transition (EMT) of LUAD cells, tumor growth, and metastasis, and was positively correlated with poor patient prognosis (65). Brain metastasis associated with lung cancer is also attributed to EMT (61, 66). We observed that patients with high *HCP5* expression had a lower prognosis than those with low *HCP5*. We found, however, that *HCP5* may regulate *HOXA7* by binding to miR-17-5p and inhibiting LUAD brain metastasis by adsorbing miR-17-5p, indicating a different mechanism of action for *HCP5* to affect LUAD brain metastasis. Furthermore, our study revealed a positive correlation between *HCP5* and ferroptosis index, suggesting that *HCP5* may promote ferroptosis. Cancer metastasis is promoted by *HCP5* through EMT in several tumor diseases (65, 67). Ferroptosis is enhanced by the induction of EMT in cancer cells (68). We, therefore, proposed that *HCP5* promotes ferroptosis and inhibits LUAD brain metastasis by upregulating *HOXA7* through adsorption of miR-17-5p.

We can speculate that dysregulation of *HCP5* expression in LUAD patients may lead to tumor brain metastasis by downregulating *HOXA7* by affecting miR-17-5p. Therefore, a decrease in *HOXA7* expression may promote the development of brain metastases. In the present study, *HOXA7* levels were significantly lower in the BNM group than in the metastasis-free group. According to GSEA, elevated *HOXA7* and *HCP5* were linked to a decreased incidence of brain metastasis in tumors and may affect brain metastasis by regulating cell membrane surface receptors. In addition, the expression of both *HOXA7* and *HCP5* was positively correlated with FPI, suggesting a strong correlation between ferroptosis and tumor brain metastasis. The study demands further investigation to explore the relationship between brain tumors and ferroptosis.

The study was limited by the small number of subjects, including clinical study, and the lack of *in vitro* cell-based validation. We plan to further validate the ceRNA *HCP5* /hsa-miR-17-5p/*HOXA7* axis' effect on LUAD BNM from basic experiments and clinical samples in our future studies.

## Conclusion

In conclusion, bioinformatics and clinical studies have demonstrated a correlation between the expression of *HOXA7* and *HCP5*. It may be possible to link ferroptosis to tumor neurovascular brain metastasis from correlation studies. Moreover, our study demonstrated that the ferroptosis-related ceRNA *HCP5* /hsa-miR-17-5p/*HOXA7* axis may play an essential role in LUAD BNM.

## Data Availability Statement

Publicly available datasets were analyzed in this study. This data can be found here: National Institute of Health (NIH), National Cancer Institute (NCI), The Cancer Genome Atlas (TCGA) Program, https://portal.gdc.cancer.gov/ and TCGA-LUAD and National Center for Biotechnology Information (NCBI), Gene Expression Omnibus (GEO), https://www.ncbi.nlm.nih.gov/geo/, GSE141685.

## Ethics Statement

Ethical review and approval was not required for the study on human participants in accordance with the local legislation and institutional requirements. Written informed consent from the patients/participants or patients/participants' legal guardian/next of kin was not required to participate in this study in accordance with the national legislation and the institutional requirements.

## Author Contributions

QC, QP, and HG performed the data collection and analyses. QC and XZ analyzed and interpreted the results. QC, YW, and XZ drafted and reviewed the manuscript. All authors read and approved the final manuscript.

## Conflict of Interest

The authors declare that the research was conducted in the absence of any commercial or financial relationships that could be construed as a potential conflict of interest.

## Publisher's Note

All claims expressed in this article are solely those of the authors and do not necessarily represent those of their affiliated organizations, or those of the publisher, the editors and the reviewers. Any product that may be evaluated in this article, or claim that may be made by its manufacturer, is not guaranteed or endorsed by the publisher.

## Abbreviations

TCGA: the cancer genome atlas
GEO: gene expression omnibus (GEO)
LUAD: lung adenocarcinoma
BM: brain metastasis
nM-LUAD: LUAD without metastasis
ceRNA: competing endogenous RNAs
DE-mRNAs: differentially expressed mRNAs
DE-miRNAs: differentially expressed microRNAs
DE-lncRNAs: differentially expressed lncRNAs
FPI: ferroptosis Potential Index
GO: gene ontology
GSEA: gene set enrichment analysis
KEGG: kyoto encyclopedia of genes and genomes
K-M: kruskal-wallis
OS: overall survival
qRT-PCR: quantitative reverse transcription PCR.
