# Supplementary material for: miR-17-5p/HOXA7 Is a Potential Driver for Brain Metastasis of Lung Adenocarcinoma Related to Ferroptosis Revealed by Bioinformatic Analysis
Source: Front Neurol. 2022 May 25;13:878947. doi: 10.3389/fneur.2022.878947 (PMC9174431; doi:10.3389/fneur.2022.878947)
Supplement: Supplementary file 1 [file Table_1.docx]

| Supplementary Table 1. Results of intersection analysis | |
| --- | --- |
| Veens | mRNAs |
| FPI-related AND BM-related mRNAs | C22orf15, KCNS2, GPR88, TBX5, MEOX2, CCL11, PACRG, FAM180A, HOXA3, UBD, SMCO3, NTF3, HOXA2, NPIPB8, PDZD9, CCL15-CCL14, AC005020.2, HSPB3, KEL, HLA-G, COL6A5, CTSG, RCVRN, TCF21, ART4, LCN6, HOXA7, CLEC4G, C12orf42, COL6A6, GPR31, TMEM189-UBE2V1, AC008764.1, MEOX1, LIPM, CD1B, NCR1, SSTR3, HLA-DQA2, AC006030.1, CLEC6A, HLA-DRB5, ACR, FDCSP, OPALIN, OTOP1 |
| Only BM-related mRNAs | MTRNR2L11, CU639417.1, MTRNR2L6, MTRNR2L10, OR4D9, AL391987.2, HSPA1L, OR5AS1, OR8B8, AL049844.1, OR5A2, OR10K1, MTRNR2L1, UNCX, NACA2, ATP6V1G2-DDX39B, OR2M3, KBTBD11, RPS27AP5, PRR35, UBE2L5, AL645922.1, TNF, OR7G2, AC073610.2, PGC, MTRNR2L8, C20orf202, AC009133.6, H3-5, PMP2, AL138752.2, TUBB8B, AC011005.1, BARHL1, HEPN1, OR6C4, C1orf146, PGAM4, CTAGE15, FKBP1C, PABPC3, OPN1SW, OR1D2, RIIAD1, CR1L, HIPK4, AC073612.1, NEUROD2, OR1A1, BPI, LRRC63, CLDN14, GPR25, OR2A1, SCN11A, RPA4, DNASE2B, AC109583.1, FSCN3, TTLL6, SPDYE17, SH2D4B, OR4K17, NLRP7, HTR5A, GOLGA8T, GRP, SSTR1, AP002884.2, CEP295NL, IL22RA2, AL360181.3, CLEC4F, ENDOU, PRR23D2, PRR23D1, BORCS7-ASMT, GABRG1, KCNH4, KCNJ9, CRYBA4, SLC5A4, HEPACAM, TTLL2, IL2, OR52N4, CST5, SGCG, AC007375.2, OR2M4, PRSS35, DAPL1, AL357673.1, GTSF1L, MTRNR2L4, RAB6C, KIR2DL4, GPR179, SETSIP, NUTM2E, AC010326.2, HEATR9, HOXC10, RTP5, MIP, SLC22A13, GLRA2 |
| Only FPI-related mRNAs | ELK3, TGM2, SRPX2, GNA15, SSPN, RIN3, ADGRF4, TIMP2, MYLK, ITGB5, PLXDC2, MARVELD1, ISLR, SEMA3C, COQ10A, ZYX, CCDC170, GPR87, NAGA, C1R, PALLD, SPARC, DYSF, CALD1, LOXL1, RALB, COL6A2, MMP7, LAMC2, TSPAN2, AXL, NPAS2, TTC7A, MILR1, COL6A1, MMP14, GAS7, SNAI2, ASPN, CPZ, OLFML2B, ACTA2, GAL3ST4, MMP11, EMP1, C1S, ARSI, C1QTNF1, PLA2R1, FHL3, COL8A2, MUC16, TSHZ3, SCARF2, CAVIN1, DSE, CDC42EP2, COL1A1, KRT5, ACTN1, CDC42EP5, COL6A3, MMP2, VCAM1, TRIM29, BGN, RHOC, SERPING1, FBN1, CMTM3, ENTPD1, KCNMB1, PCDH7, CCDC80, CALHM2, HTRA3, AEBP1, COL1A2, THY1, SCGB1A1, PLEKHO2, NBL1, LUM, PML, LAMB3, CAVIN3, PLSCR3, UNC5B, C1QTNF6, COL3A1, CLEC11A, PDGFRB, SGCD, NOX1, ADAMTS2, TMEM119, FRMD6, DCLK1, MXRA5, EMILIN1, PRKG1, TAGLN, SLC22A4, SPATS2L, CD4, PRSS23, LAMP5, TNS3, COL5A1, RAC2, COL15A1, CHST15, LRRC15, VASN, FN1, ACTG2, THBS2, MSRB3, WDR38, PRKCH, TEKT1, COL10A1, C20orf85, OLFML1, LIMK1, DAPP1, GNAI2, PLSCR4, PPP1R18, EPHB2, BICC1, EXT1, MTERF2, HTRA1, CDA, PRR5L, ALOX15, IGFBP7, ITGB4, PLA2G5, FSCN1, ADGRA2, FSTL1, MYO1E, HEPH, TMEM200A, ADGRL4, ISM1, BEND6, FBLN2, CD109, ENKUR, MRGPRF, KRT16, ITGA1, C1QTNF5, DACT3, OMD, ITGA11, RAB11FIP5, PTAFR, SLC2A9, CTSK, COL5A2, ANO1, ENG, FLNA, GPX8, KRT19, CD248, TGFB1, IVL, CXCL12, AHNAK2, MMP10, FAS, CILP2, CFH, PLEC, GPR1, GLT8D2, RRAS, KRT6A, PLAU, FBLN1, TMEM204, CPXM1, TNC, C11orf88, LMO2, PRRX1, ACSL4, CSF1R, TNS4, CDH11, FAM216B, C1orf87, ZFP36L2, FYB1, LAT2, PLIN3, TUBA4B, ZNF474, UNC5C, LMCD1, SLC39A11, EFNB2, SKAP2, LGALS3, ENO3, PMP22, NETO1, GNA12, ARHGAP10, ANXA8, RND3, MAP3K5, MAFB, TNFSF12, SLC16A2, LXN, TNIP1, TTC9, TGFB3, ITGB3, TM4SF18, LAYN, VSTM2L, FMNL1, CSKMT, SULF1, EMP3, BMP1, ROPN1L, WBP1L, TBC1D22A, CYGB, DCN, COL8A1, RHBDL2, ETS1, CRYBG1, ADAP2, TNFAIP8L3, ACTB, RHBDF2, PLCD3, CST6, ECM2, S100A9, NCF2, NKD2, VSIR, TLR1, COL18A1, SP100, LOX, BHLHE22, TMEM109, CSDC2, DAB2, COL14A1, POSTN, MXRA8, EFCAB1, ATP10A, AHNAK, STARD8, TAX1BP3, DST, WIPF1, SSC5D, ANXA8L1, CYTH3, GJA5, VOPP1, TP63, DBNL, SELPLG, CAV2, ADAMTS12, NALCN, ARL6IP5, RASGRF2, GASK1B, PAN2, SYTL4, XDH, MORN5, MSN, CARD11, MRPS36, CDHR4, PIP, MSC, ITGAV, FHOD3, TMEM190, ANXA2, VCAN, INPP4B, GPR176, TGFBI, CD82, PTGS1, MAF, FAP, COX7A1, IGFBP4, FAH, CAB39, LAPTM5, LGALS1, VEGFC, APOBEC3C, MDFIC, TRABD2B, LMOD1, GYPC, MITF, VIM, TBC1D2B, C12orf73, ABI3, ATP10D, GIMAP4, GKAP1, PMEPA1, SNX22, GJB3, RHOG, SH3BGRL3, SIRPA, CLMP, DKK3, LRP1, CILP, TNFAIP8L1, ST3GAL2, ITGA2, CSF2RA, ZCCHC24, RGL3, MAML2, PDLIM2, LAMA4, AFAP1, GLIPR2, RAB8B, IGSF21, C11orf97, VTI1B, WNT7B, OMG, LRRN4CL, FLI1, RNF135, CXCL11, TPM4, TMEM51, PDLIM5, LEAP2, DTHD1, NEK6, CDK14, CACNA1C, GJB2, F13A1, NEXN, SPON1, DOCK10, PDLIM7, DHRS9, PDGFB, CNN1, IL20RA, SAMHD1, RNASE6, TMEM140, RAB31, LAMB1, MYL9, CHST9, ITGA5, ARNTL2, RARRES1, EPB41L2, CAPSL, JCAD, EHD4, PAPLN, BBS12, NAALADL1, DTX2, LSP1, FGD5, ITGB2, SAA1, PPARD, TMEM106A, ABCA12, LRRC32, FZD1, MTMR7, IFI16, HIC1, ANTXR1, MN1, HAVCR2, RGS22, IDI1, WNT10A, C1orf194, LACTB, GALC, ZNF185, CD93, MOXD1, TUBB6, MARCKS, LTBP2, PTGIR, TICAM2, TSPAN19, PRR29, HSPG2, MYEF2, GALNT15, C3AR1, PDCD1LG2, MYH7B, ANKRD66, SMCO2, RGS10, NOX5, CFAP52, PRDM6, TMEM212, SDC3, FMO1, STEAP1, ADGRV1, ITGA3, FGF1, TGM5, STOML3, ANGPTL2, COL12A1, RAP2B, STK10, ZFPM2, MYH11, MOB3B, YWHAH, CALHM5, SP110, ARHGDIB, PDGFC, STK17A, VGLL3, RASD2, NID2, TNFRSF6B, SH2B3, GGT5, TGFB1I1, PDE2A, ITGA8, SLFN5, CYP2F1, LYSMD1, KRT7, DXO, SPSB1, GBP6, CCDC3, TLR7, CCN3, ZNF521, CFAP77, SS18L1, FZD3, CNBD2, GNG2, LRP10, SYDE1, HEG1, ALOX5AP, TTYH3, FIBIN, GIMAP6, KIAA0930, LPAR5, EHD2, ARHGAP18, MCAM, TTC32, EBI3, JDP2, RNASE2, MAP7D1, G0S2, TMPRSS11E, TTC29, DCBLD1, LAIR1, SEMA7A, RSPH1, GXYLT2, COL17A1, CAPG, MS4A6A, PSCA, DKK2, ANXA6, BHLHE41, CELF3, PCED1B, MAX, LRRC46, KRT17, LCP1, A4GALT, TREM2, CAMK1G, GIMAP2, GBP2, SLC7A7, JAK2, SPI1, FXYD5, SH3KBP1, ITGBL1, TBXAS1, RASL12, RGS19, EHD1, AKAP14, GJB5, CDKN1A, GUCY1A1, PROCR, WSCD2, PIK3AP1, TNFAIP6, PEA15, CFAP53, SEC11C, SFXN3, EDN1, HAPLN3, PLPP4, SLIT3, ENOX1, DLL3, PODNL1, ECT2L, SAA2, AP1B1, TMBIM1, TMEM248, JPH2, DRC1, SLC38A3, AMPD3, CRISPLD2, MFSD11, EDNRA, CNRIP1, VTCN1, CCDC113, CFAP126, SH3PXD2B, ERICH3, C16orf71, DNAH9, SECTM1, PLVAP, CLDN16, KCNN4, CD163, PTTG1IP, SAMD9L, ANXA5, SMIM3, BCAR3, CXCL6, LGALS9, MAP3K19, FGFBP1, AMFR, OR10AD1, GFPT2, SNTN, ST6GALNAC5, TMEM52B, NAALADL2, ADORA3, THBS1, MOB3A, STEAP2, HLA-E, WDR78, SYNC, SLC66A3, BCL2L13, LRRC17, GM2A, COL11A1, CSTA, SPHK1, FAM81B, GSDME, TNFAIP8, RASA3, OLR1, OXTR, LYPD5, FMOD, STAT4, BRD8, APBB1IP, DIPK2B, GAS1, FABP6, ZEB2, PLSCR1, PLB1, APOBEC4, SFRP2, TIMP3, NCS1, PGM2, NWD1, IL21R, KIF22, MYH9, A2M, CDC42EP1, SAMSN1, LCP2, C9orf24, TMEM41B, IL20RB, BPIFB1, SCT, S100A16, TSPO, RSU1, RHOJ, SLC16A3, MDFI, CASS4, SFRP4, TMEM87B, CFAP73, TSPAN9, FEZ1, PLN, PLXNB2, APLNR, HEBP1, IFNGR1, CD33, GSTK1, KCNK6, KLB, KCTD11, CLEC2B, FAM20C, TEX26, CPED1, BBOF1, ITPRIP, ADAM12, AC087632.2, FAM220A, BIRC3, SHLD3, SLC9A7, PXDN, OAF, KRT6C, RAB27B, RAB3IL1, COL4A1, NOX4, RIN2, CNN2, TCAF2, ASCL1, WDR1, ANXA1, TNFRSF1A, HIF1A, CLMN, AC007906.2, FAM183A, C1QC, FPR3, ADAMTS16, C1orf54, HCK, SH3GL1, TNFAIP8L2, IP6K2, C6orf118, CX3CL1, TMEM86A, MAPK11, TYMP, ART3, CBLN3, MNDA, MEIS3, EVI2B, FLRT2, ANO6, HOMER3, GALNT10, NDUFAF6, PTPN22, LY6H, ZNF620, KRT81, SELL, KIAA1755, CACNG4, CLIP2, ITGAM, PLAT, ADORA1, KLHL5, NTN4, C12orf76, CTSO, FCGR3A, WDR63, SLC9A9, MRTFA, PDZK1IP1, GALNT5, GLIPR1, P2RX7, CD99, KYNU, GRIN3B, DEPP1, GPR132, FSTL3, CD37, PPP2R2B, SULF2, TNFSF13B, SPON2, CCM2, C20orf144, ABLIM3, RNF144B, NHLRC4, SIGLEC9, DCDC2B, CCDC71L, COL4A2, RTL3, PLXNC1, VDR, EVA1B, SIPA1L1, DPYD, CD300C, MTSS2, KCTD12, EDIL3, CAPS, RASSF2, SYNPO2, OGN, NTAN1, ZNF423, SQOR, PIEZO1, GPC1, FHL2, CATSPERD, TTLL1, CFAP43, IL32, FMO3, FNDC1, FAM166B, IL4R, PCDHGA9, C4orf47, FPR1, PDGFRA, FMNL3, FILIP1L, DUSP18, GIMAP1-GIMAP5, DOCK2, RASAL2, BMP2K, LATS2, HOXA5, CASP4, RUNX3, PLEKHG1, TACSTD2, GIMAP8, KIAA1958, DKK1, VWA3B, CHPF, PRELP, RSPH3, CORO2B, PRSS53, OSBPL3, CD86, LRRC25, PLD4, TNFRSF1B, TIGD1, HLA-B, ZMYND10, SIRPB2, SVEP1, LYN, TNN, ARPC2, DCBLD2, PDXP, HSD11B1, SH3BGRL, CLDN1, CCDC14, MYOF, JAK1, TCF4, LRRK1, HS3ST3B1, ARMC3, LITAF, SPATS1, DPT, EPHA2, MAN2B2, LRRC18, NME9, SRD5A3, CCR1, KCTD17, TNFSF4, SLCO2B1, HYPK, AKR7A3, CNGA4, UQCRHL, TLR3, AFAP1L2, PLAUR, TIE1, NABP1, FEZ2, SAA2-SAA4, SEPTIN8, TRPV2, CFAP299, SGMS2, FAM131B, RAB26, NT5E, AIF1, CX3CR1, KCTD21, NECAB3, TYROBP, PTPN14, APOL6, NPR3, ZBTB26, NR2F2, FLVCR1, CST7, CARD16, NFATC1, SLC15A3, XIRP1, PTPDC1, TMEM232, DAW1, GALNT3, MFAP2, IL17RA, HOXA4, TMEM92, MARCHF2, ROR2, IMMP1L, TMEM71, MYO3B, PLEKHN1, GUCY1B1, AC090360.1, CROT, PROM2, KIAA2012, LRRC71, ARF6, GOLGA8B, VCL, MMD, C1orf189, IL7, IL27RA, KRT15, ARPC1B, ACTN4, MATK, FBXO32, CARD6, CABCOCO1, PERP, PPP1R42, RPS6KA4, SUGCT, PLA2G15, IL18, RPAIN, KRT86, IGFALS, LUZP1, IFI35, KCNA5, CD34, STAT5A, SLC24A2, ARMC4, TSPAN15, KCNE1B, SNAI1, BCAS3, CTHRC1, SH3RF2, EFEMP1, ZNF251, VSIG4, WFDC6, RERG, GPR34, MAP4, LY6G5B, VAT1, SIGLEC14, CFAP65, FCER1G, FGF7, NCKAP1L, IL3RA, SAMD4A, MID2, PTBP2, GREM1, CAV1, CPXM2, WAS, STARD4, TMEM200B, TMEM154, PLD5, SLCO2A1, AFAP1L1, GPR182, MFSD1, TMEM102, RAB43, EGFR, CCDC33, COPZ2, APOL3, OGFRL1, TMEM127, SERPINB3, CD14, B3GNT3, PRKCA, ABHD15, CH25H, KCNK13, TWF2, RSPH10B, BCL2L1, BOC, NLRP12, RAB3IP, MSI1, NEURL1, CCN4, FKBP5, AMIGO2, PTGER4, SLAMF8, TSPAN18, METRNL, ANKUB1, CCDC8, FAM78A, ZG16B, LIMS1, SAMD9, NAMPT, IL15RA, BATF, BACH1, RELL1, S100A10, TCTE1, CD59, LAMA2, ZNF469, LIPE, SNX10, C1QA, USP25, TCN1, SPARCL1, NLRP3, PLEK2, CSK, UGT3A1, PALMD, ADAM8, TAGAP, ADGB, IFIH1, CFAP206, RTTN, UNC13D, NFKB1, DLGAP1, CRTAP, CASP1, SEPTIN4, BNC2, LAIR2, C1orf158, SYNPO, PHLDB2, PHC2, SCN2B, SEPTIN9, LTBP1, CSF2RB, PSEN1, APOBEC3F, GJA4, SNX8, MYO5A, CD53, ZDHHC14, P2RY12, FUT7, AP2M1, ALOX5, JAG1, FOSL2, FAM124B, ARHGAP25, TPSAB1, CRYBB1, LY86, SEPTIN11, DDX55, SPRR1B, COL5A3, CEMIP, MELTF, MSR1, CYTH4, SH3BP1, LY6E, PODN, INPP5D, CLEC7A, CFAP58, ISLR2, MAP3K7CL, FCGR1B, TUBA4A, KHDC4, RANBP3L, FAM24B, CSF1, PLAAT4, MYO1B, FOSL1, KERA, FCGR1A, MAP7D3, MFGE8, PDXK, CTSC, RGS18, LAMP2, CSTF3, ECRG4, ZEB1, HSPB2, PIP4K2A, CRLF2, CLIC2, MCTP1, CFAP161, PZP, MAMDC4, AHR, PALD1, STUM, PSTK, PDGFD, TMSB10, DDIT4, CNGA1, C1QB, C1QTNF2, MYO1D, ZNFX1, GPR84, CCR2, CPD, NXPH3, SCML2, ADCK2, TIA1, ACOT11, HSD3B7, PLA2G4C, NEK1, DNAI2, SLC2A10, DPH7, CDH3, CST2, MRC2, CCDC69, RARA, ZFP36L1, LY6D, CCN2, CKLF-CMTM1, DNAAF3, MARCHF1, AGRN, NR0B2, MYCT1, TRAF3IP2, GBP1, APOBEC3G, SNX20, KIAA0895L, DIO2, CCL21, LMNTD1, SYT8, ECSCR, ZBTB38, CCDC183, FBXL7, CAP1, PRR3, SVIL, GLP2R, SMARCAL1, RGS5, C1orf162, RBM39, ARHGAP15, RCN1, CYB5R3, EPHB6, SUMO3, DACT1, HCN3, HCLS1, SIGLEC10, SLC1A3, FXYD6, LYPD3, KRT14, BID, ERG, TPST2, CERCAM, MST1, TCTE3, DDR2, SLCO4A1, TSPAN33, BCAR1, ATP6V1C2, EFNB1, GNL3, NSUN6, NELFCD, ANTXR2, KLHL4, GIMAP1, WWTR1, NCF4, LIMS2, NFE2L3, ZNF692, C2orf73, CFAP57, SYNDIG1, CARHSP1, C11orf45, SFN, PXN, TRAF1, AOC3, PDLIM4, MAP4K4, RGS3, MROH9, SIDT1, GVQW3, RCOR3, ANKRD35, MSH5-SAPCD1, IER3IP1, ST6GALNAC4, MYD88, LOXL2, RASGRP1, S100A2, APOL2, HMGCR, CHST14, FLNC, ZNF891, FOLR2, DPY19L1, RNF125, CLTB, PIK3CG, C1orf141, EBF1, STX11, AOAH, C4orf36, AC134669.1, NFAM1, MAP6, SERPINB9, CEBPZOS, LACC1, GNG12, RET, CTSB, C7, SUFU, TMEM255A, S100A11, SLC10A5, CCL19, ARID5A, MFAP4, HSD17B7, IKZF1, DENND6B, IQUB, KCNJ11, PCOLCE, ANKRD22, PARP12, TMEM45A, SOD2, PARVB, MSH5, BBIP1, TEKT4, PLAAT2, DDX58, MEF2C, MMRN2, FOXP3, CMKLR1, SUSD6, HCST, FNBP1, EEPD1, DDC, CYB561A3, SAPCD1, RARRES2, LYPD1, TSKU, SS18L2, LZTS1, KCNRG, SYCP2L, MAP1A, PPIL6, CD200, OPN3, SOX18, CYYR1, WNT2, SCN3A, UBE2L6, PECAM1, FGL2, FAT4, SERPINE1, GPR141, NELL2, RSPH10B2, KLHL2, KATNAL1, IRF5, EDRF1, SYTL2, ZBBX, CARMIL3, MOB3C, ROBO4, ORAI2, PRKCB, NSMCE4A, RAP1A, CD74, CCR5, TLN2, SLC31A2, GLRX, JHY, HSPB2-C11orf52, ALDH1A3, IL16, CCDC74A, NDN, LRRC4, TMCC3, IRF8, SELP, FCGR2A, NID1, CAST, CERKL, STOML1, C11orf98, PEBP1, TRIL, CHI3L2, HLX, PREX1, DYNLRB2, TSPEAR, GIMAP7, RCSD1, CRAT, CLEC1A, INSM1, C11orf68, PCDHGC3, SH2D3C, TES, SNX7, TTC13, TRIM34, MS4A4A, PRR15, HR, CASQ2, HLA-A, RUBCNL, PTGIS, CLEC10A, SLC35G5, ARHGAP20, BTN3A2, TP53I3, MT2A, RIN1, NEK10, LAD1, TRIP6, FJX1, CEACAM16, TENM4, PLXND1, ARHGEF6, NRP2, SEC61A2, RFLNB, HPGD, NPTN, EPSTI1, TBXA2R, XKRX, TLR4, LMNA, STRC, GJA1, IQGAP2, ARL2BP, TPSB2, NFKBIE, ASAP1, TRAF3, INHBA, LHFPL6, IFIT2, SPN, HS3ST3A1, EHF, MYADM, NAV3, NMI, RIMKLA, FLII, RAB33A, RFFL, SYT12, WARS1, RAET1E, TMA7, CAMK2N1, CALCB, IRF2, DOK2, CYBB, EPHA1, MRRF, HSPB7, CCDC6, DUSP14, CCDC150, CD68, GDF9, AC013470.2, PRRX2, CLIC4, VRK2, ITGB1, GMFG, PGR, PDE4A, BTG4, BMPR2, AGAP4, TRMT10B, EGFL6, SRRM5, RIPPLY3, BPGM, CD40, NAPB, RNF183, EHD3, GAB1, NECAP2, OAS2, AKAP6, PRPF3, CENPV, EVI2A, CORIN, ZNF248, MANBA, PKIB, OLFML2A, CLASRP, COTL1, GAPT, EXOSC8, ST6GALNAC2, DRD5, F2R, ELN, PILRA, S1PR1, MMP9, CACNG6, CHSY1, CRIM1, RAB32, SPECC1, PCDH18, ERO1B, DGCR2, TNFSF8, PLEK, PPL, MBOAT2, GLIS3, PICALM, DSG2, KLF10, MLLT11, SERPINF1, ADORA2B, SH3D19, FHAD1, CLCA2, FNDC4, ECE1, SBDS, TDO2, SEC14L3, TMEM229B, GRN, NOD2, PPP1R3G, PLAC9, CACNA2D4, CPVL, SPRY1, HHLA3, INF2, EFEMP2, EDN2, ACTR2, CGA, KIF13A, ACVR1, NOTCH3, CCDC17, SCARB2, INAVA, SPDEF, OXSM, COL16A1, SEC14L2, ZRANB2, PCDHGB7, TOM1, CCDC154, TPK1, PCDH12, UACA, GBP4, ARHGAP31, SCIMP, TMEM106B, IL2RA, PCDHGA12, CSGALNACT2, MBNL2, LDLRAD3, DSC2, PKD2, UBE2Z, FBLIM1, LILRB2, HES2, LDB2, FBXO16, RRAGC, CHTOP, COMT, JAZF1, PHLDA3, KRTAP5-10, ZPLD1, GLUL, EREG, AP1M1, GRAP, ZHX2, PRNP, CCDC78, COLGALT1, CCDC146, SMN1, RSKR, RASGRP4, ANXA4, RAC1, HLA-DPB1, TNFRSF12A, GAS2L1, TNFRSF21, KHDRBS2, ZFYVE1, SLC29A3, OGT, KLF8, GATA3, FNBP4, PTPN9, RAB23, KIF5C, PGBD5, ANO9, CXCL1, EYA1, NOTCH2, THOC1, GLG1, NGEF, PHEX, RIPPLY2, LASP1, UNC45B, HAMP, AGAP6, AVL9, PCDHGA10, SPAG6, ATAT1, SSH1, ZBTB47, CLK2, RTN1, NR6A1, UBE2E3, C7orf31, ODAPH, IL12RB1, MSS51, OR2I1P, CALHM6, STAC, COLEC12, VGLL1, EXOC6B, GPR75, AHI1, RSPH4A, SGPP2, ABCG2, LEXM, FBXO36, PLEKHO1, NCEH1, DDN, MEGF10, C5AR1, FOXO1, CEACAM21, PNISR, PLEKHA2, CTSS, FERMT3, MXRA7, MEDAG, SYP, BCL6B, CALCA, DSCAML1, NDUFAF5, MGP, SASH3, RSAD2, ZMAT4, TCTEX1D1, GFOD1, OSCAR, UBASH3B, CCDC102A, ELFN1, LEPROT, STBD1, FADS1, PEX26, ABCC8, PID1, GNG3, TLR8, LHX4, TANGO6, ESS2, BST1, PCP4, CYP2R1, PLPPR3, IL2RG, SH3BP2, PAXBP1, FCGBP, MPZL2, SEMA6B, CHKA, RIPOR2, VNN1, CCDC60, C7orf57, ARAP2, TMEM120B, NINL, SLA, ALPK1, IL18R1, PIP5K1C, FOXS1, MT1M, PDLIM3, RBIS, RARG, ABI3BP, BTN3A3, CCDC173, CASP10, MFAP5, VAV1, GATA5, COMP, IKZF2, PINK1, TRIM6, ABCA6, SECISBP2, RUNX1, HK1, SMPD1, FAM13C, STEAP3, CD180, SLC25A33, ITPKA, ADPRH, PEAK3, ANXA3, CTSD, GPR65, TTC6, GPC6, IRAK3, PLEKHM1, FANCD2, PIEZO2, CYP2A13, ELF1, DYDC1, ZBTB5, TRIM47, APOA5, TIAM1, EPYC, CYB5D2, ARFGAP3, UPP1, KIRREL1, THPO, KIF17, HLA-F, TAF6L, DNA2, TRIM14, FAM47E-STBD1, MATN3, BIN2, CDK15, PTPRC, CFAP45, GBP3, SIGLEC7, FAM102A, FMO2, PTPRJ, LILRA1, CDH13, CCND2, LYL1, PLAAT3, IL1R1, TMEM273, CYS1, VGLL4, AGTRAP, SLA2, SRSF6, CSPG4, AP2B1, RABGGTB, WSB1, NYNRIN, AL020996.2, LRRC8C, ZNF385D, GPT, IGFL3, HIBADH, ITPKC, TYRO3, IL10RA, GTF2H2, ZNF124, ABHD4, DOCK8, GPR68, TCEAL7, TM7SF2, GPR153, STAB1, GSN, ZNF397, TMEM156, OPTN, HAX1, RAB3A, ACVRL1, GHR, GNAT1, CEP126, CCDC90B, S100A3, CD84, ZNF738, PET117, SH3GLB1, OR13J1, UNC13B, DDX60, VASH1, ACBD7, CORO1A, DPYSL3, DNAH12, SRD5A1, PTPN21, SERPINA1, AL035461.3, ARHGEF3, CSMD2, LBH, B3GAT2, LCN15, CCDC81, BEX1, PARVA, ZBTB46, ABHD12B, RSPH9, MBP, POGLUT3, ODC1, DRC7, PTPN7, CANT1, FASTKD1, IL2RB, RIPOR1, RTN4, DUSP5, TRIOBP, C19orf38, EIF4H, SMIM26, CMTM1, FAM83A, RBMS1, KCNH6, KSR2, MICAL3, IL1RN, PPFIBP1, EPS15, C1RL, TSPAN1, MAL2, ZNF726, ZBTB37, INHBC, PIM1, TRIM8, HERPUD2, ADAM9, HSF4, CCDC89, PTGDS, EFHD2, PRXL2B, GABRD, ZSCAN16, DNAI1, NEK11, TMEM145, TMIGD2, SMOC2, DISC1, HLA-DPA1, CCN1, CCR8, CCR7, ARIH2, FAM229B, S100A8, HAAO, KRT80, BCL2A1, STK17B, ZNF572, GLIS2, ROR1, SAV1, FAM240C, RNPEPL1, FUT8, NAAA, SPEF1, SYT16, LRRIQ1, POLR1C, CFAP74, MSX2, SPATA4, PLTP, HLA-DRA, LRRC23, HACD4, CCDC66, APOL1, MASP2, LST1, ARHGAP30, GTDC1, CST1, STAT1, TTC16, TRIB2, TIMP1, TMEM255B, ADGRE2, ATG4D, LETMD1, PLPP7, LHFPL2, CCNL2, RAB27A, ITPRIPL2, ENO4, MMRN1, SMO, ARSB, SCG3, GNA13, GAS6, GADD45A, RHOD, TLCD4, GALNT14, CFAP61, ZNF514, FAM107B, SAMM50, TLCD4-RWDD3, DNAH2, HES6, TCN2, NUDCD3, TDG, CD276, CCL22, PARD3B, TBC1D9, METTL3, TGM4, ZNF366, CCL17, AC068580.4, MRPS26, GH2, SLC40A1, CSGALNACT1, E2F5, PDPN, LIPH, SLC29A2, ZBTB8B, ST8SIA4, STRA6, SMG6, PGLS, FIGNL2, PRR16, CLEC4A, SAMD15, PRAG1, CLEC5A, CYP7B1, LMF2, IGFBPL1, PGM1, MRNIP, MTHFD2L, SPNS3, FDFT1, FMN1, MLKL, SRGN, RAET1L, CPA3, SLC39A5, TAS2R20, HYLS1, ITGA6, CD80, TRIP10, CMPK2, C12orf65, MAT2A, TWIST2, GJA9, PCDH1, CD70, UBE2F, LILRB4, NAGPA, HIVEP2, CD200R1, GPR4, CD1D, ATG7, ARHGAP23, HHIPL1, PPM1E, TMEM159, WASF3, SCN7A, COX7C, CAPNS1, PRICKLE1, CCDC103, ELL3, LRCH1, FAM3C, RAI2, SLC25A24, MRPL48, CHST11, TMEM132E, UBC, TRIM50, RASGEF1B, CYP24A1, GPNMB, EPM2AIP1, CDK3, CYP2S1, SLC14A2, SLC26A6, MMAB, TAP1, SLC30A1, TMC2, ELF4, ROS1, DES, NFE2L1, PHETA2, PLPPR4, KDELR2, CD209, MT1F, ZBTB7A, ZNF782, CA5A, ZNF10, NLRX1, P2RY13, GDF5, CXCL10, ENTPD8, IRS1, NKX3-2, CDS1, CD44, IKBIP, SOCS1, TERT, PLA2G6, LTA, PTPRO, UCKL1, DUSP3, METTL17, CBFA2T2, PRMT8, STXBP5, HLA-DMB, KIAA0040, SLC12A8, TSEN2, KCNC4, RECK, L3MBTL2, RAB38, TWIST1, SEC14L1, CHST3, DHRS7, POLR2H, LY75, SLIT2, C11orf91, CCDC96, CCDC39, IMPG1, PNPLA7, RBMS2, EVPL, MINDY4, SNX9, SLC10A3, GPR183, S100A7, IL4I1, TIGD3, FUT4, NFATC2, ACAN, SGCA, ADAMTS7, ZNF670-ZNF695, TRPV1, C18orf32, N4BP1, MMP13, ENTR1, SLC23A1, IL5RA, NXN, TINAGL1, TTLL12, SRPX, NCK1, PPARG, TK2, TNFAIP3, AMT, KRTAP2-3, MED15, KIAA0513, ABCD2, ZSWIM4, HOXA6, STX16, SH3TC2, N4BP2L2, RNF141, MR1, AIG1, C1orf100, VGF, SLC10A7, AC074143.1, CRIP1, VWF, ANKH, ARHGAP6, APOBR, DCDC1, ACKR1, CCDC84, CAPZB, AC069503.2, CATIP, PRELID3B, PLXNA1, TMCO3, PNMA6F, SEPTIN7, SEL1L2, FXYD4, TSPAN5, OTUD3, ADAM19, CACNA1F, ITGA4, SKI, PERM1, ZDHHC18, SLC38A5, SIGLEC8, RELB, MTPN, IDS, REM1, TNFRSF9, PAPSS1, SLC35E4, ABR, CAPNS2, TGFB2, SERPINA10, CCL14, IBSP, SMIM10, PRPF40B, TNFSF10, TRIM72, KCNH8, SUN2, ENPP2, TBX10, MEAK7, PEPD, PTCD3, CCR6, SCAI, PABPC1L, BMP15, CNTNAP3, PAM16, ADAM28, MYO1F, TMEM262, HERC6, FASTKD3, CCDC65, BICD1, FCGR2B, CACNA1D, SEMA3D, UBE2D1, FRZB, CYP1B1, LRRC6, C1orf127, CETN3, YPEL2, C8G, ENOX2, ZNF319, CCN5, MET, UMODL1, PRDM8, CCNG2, AZGP1, SRRM4, TMEM121, CFLAR, APBB3, C6orf136, PLEKHG6, OPRD1, CUEDC1, CDC27, MDM4, CRTAM, LTO1, ACYP1, AGAP5, NCK2, CHRFAM7A, PIK3IP1, PGBD1, SPRR2F, STARD13, PLS3, POLDIP3, TTC22, ZNF789, ADARB1, NCCRP1, CLECL1, AC012184.2, NDRG2, OPRL1, ARSJ, TSC1, TNFSF13, CCNL1, CALM1, PSMB9, HIGD1A, ZNF169, SLC35G6, HSPA12B, KLHDC7B, SLPI, MCM5, ATOH7, AUTS2, DNM3, PRPF39, AK8, APOA1, TTC25, GINM1, ANGPTL3, MCC, UBXN6, POP5, TPRN, UBE2H, SLC25A20, REV1, SLC16A13, C16orf74, TUBA1A, SPDYA, S100A6, RCBTB2, ANAPC7, GIMAP5, CPNE1, GNAZ, CWH43, IQCE, TBC1D3H, ZNF239, MGLL, LRRC8A, HMGA2, WDR49, VANGL1, GNB4, MAN2A1, TFEC, GAS2L2, S100A14, TRAM2, CPNE2, MCFD2, HIPK1, DFFB, FLT3LG, APBB2, PDZRN3, GAA, CSAD, GNRH1, GSTM5, CFAP100, TRPV4, TPST1, ALKBH2, FOXO3, IL34, ODR4, VPS37B, PRRG4, CNP, TAP2, MAP3K14, KCTD1, KLHDC9, RRAD, TEX45, ZNF695, ZNF141, ITM2B, MCUB, MGARP, RBMS3, EMILIN2, PLPP3, PPFIA4, LCN12, JUNB, LAP3, MAP1B, C15orf61, ISOC1, RBM43, ZNF664, ARHGAP1, CHRNB2, PLBD2, TBC1D1, HIVEP3, SETD7, PIK3R6, LYNX1, NPIPB6, CALCRL, PHETA1, CALU, TSR3, PYCARD, CNIH3, WNT5B, DOK5, B2M, TMEM185B, LRIF1, CAPN2, CDH5, DCHS1, FAM184A, NDUFAF4, HLA-DOA, MT1X, ABCA4, NAGK, LETM2, MYCL, GNAI3, CRIP2, CORO1C, DAPK3, FOXA3, LPXN, SAXO2, BAG3, HMOX2, PFDN6, WDR74, HMOX1, ADGRE5, LY96, SYNGR2, SAMD3, ZNF436, ALG12, PGM2L1, SAMD14, GTF2H2C, P4HA2, INKA1, PADI1, REEP6, HLA-C, PLOD1, FAM114A1, RGL1, LPAR6, IL7R, CASC1, DSCAM, PPP1R27, IL17D, RASA1, BBS9, POU6F2, PADI2, ESYT2, ENAM, LGALS12, POLI, TCERG1L, IQGAP1, RBP1, CXCR5, SMIM14, PARVG, ZFC3H1, CHP2, EBLN2, HTR1D, BLVRB, FRS3, TRANK1, ERAP2, BCL9L, WDR5B, IGSF6, BCCIP, PLBD1, NHSL2, VSIG10, CDK6, MICALL1, TSC22D4, IL15, IGFBP6, NEK5, ACTBL2, STYK1, ACAT1, SPATA18, PDP1, ADAMTS1, IRX2, TMEM26, FAM110D, NPTX1, BLVRA, SERPINB5, INPP5J, TEKT3, SRSF11, LAMA3, TOX2, CLEC14A, C9orf116, C16orf46, BEND3, IGFBP3, GNAI1, SERF1A, SPOCK1, AC011448.1, PSAP, CEP83, PVRIG, PTP4A2, BCL10, RBM14-RBM4, C1orf43, GPD2, CTTNBP2NL, P2RY6, TLN1, TAPBPL, SMAD3, ERMAP, DHH, ARL4D, MRC1, LRRC59, SERPINE2, EPOR, H6PD, MCCC1, STX16-NPEPL1, REM2, NIPAL4, CDC42EP4, EFHB, DEF6, NFATC2IP, OGDH, RAB8A, NRBP1, MAPKAPK3, IRAK2, SOX7, SDCBP2, SNAPC2, NBDY, SERPINH1, TSNAXIP1, RSPO3, CYLD, STMND1, PFKL, AL049839.2, PCDHB3, BCR, IL13RA1, SLC35D1, PARP9, FZD7, GPR152, GALNT1, MUC5B, TLE6, ATP8B3, SNRPE, ARHGEF39, ERAP1, ZSCAN30, STING1, NNMT, TMEM170B, AL669918.1, GJB4, DYNC1I2, LUC7L, NRIP1, C1GALT1, CHCHD1, FUCA2, SNRNP48, COX7A2, SPIRE2, ADAMTSL2, SLAMF6, FGF9, MARCO, PI4K2A, TMEM104, ZFAND1, USP21, TCF24, FAM187A, CLIP3, GOLGA8A, C6, SLC25A18, LILRA4, CDKL1, JOSD2, BCO2, BANF2, IFT27, KCNE1, ITGAL, PYGB, CEP85, NBR1, ANKFN1, FBXW12, AC022400.5, GOLGA6L9, FBXO15, WDFY1, PPM1F, NAGLU, HPGDS, CBLB, SLC11A2, PRMT2, WFDC1, DGCR6L, STAB2, JAM2, CEACAM4, NIN, CRIP3, S100A1, STMN3, RHOF, IL17B, ORMDL1, ITGB8, SELENOK, H3-3A, FCF1, TXNIP, PNRC1, DDTL, BTN3A1, SPATA21, GSDMC, IGDCC4, PGBD2, ZNF33A, ERO1A, SLC6A9, TBX21, OR4C6, ZNF354B, POLR1D, KDELR3, FAT2, CC2D2A, CLCF1, KRT6B, STPG3, OR1M1, PAMR1, SOX9, DHX58, ZNF438, IDO1, HSPB6, NKAIN2, EPN3, ARL4A, FAM167B, TIMP4, RNF24, CD3E, WDR81, TMED7-TICAM2, USB1, APOC2, FLACC1, BEAN1, HOXA10, NPIPB9, PNN, SH2D1B, TGFBR2, TOMM20, SEC23A, TNIP2, SLC52A3, SYPL1, CCDC74B, IFFO1, TMC1, MAPK10, DNAH7, STXBP5L, CLSTN1, GZMK, SOWAHD, FADD, SMAD9, POLR3F, CUX1, ANK3, FAM3D, LDLRAD1, KCTD5, GMIP, FTO, ZNF195, CAPRIN2, OLFM2, RSRP1, AMOTL2, TOMM6, SELENOV, KLRF1, PACSIN2, NGFR, HECA, HSDL2, CFI, CAMK2A, P2RY14, IL17REL, PXDC1, XKR7, NFE2L2, CYBRD1, PTGER2, ADGRF1, C22orf23, ITGB6, MYSM1, SHANK1, PLAC8, GALNS, TMEM47, B3GALT4, PRCP, LBX1, MFNG, UBE2T, TRIM46, SPRYD3, MYL3, SERTAD1, TRIM49D2, ACTL6B, HEPACAM2, S1PR2, AC010132.3, MCTP2, PPP1R13L, RASAL3, ICAM3, MTCL1, WDFY3, UFD1, CFAP47, ADAMTSL4, SMARCA2, EPHA7, FGL1, AC010255.3, CCL2, FNDC11, HLA-DQB2, LARGE1, PAK1, CCNB1IP1, TNFRSF11A, CFAP157, PACS1, KLK12, ST3GAL4, PIK3R5, TNFAIP1, DNHD1, DNAJB4, CYREN, RBFOX2, SLC35F6, TMEM183A, PSMF1, SPDYE6, OSMR, QKI, MS4A18, GPRC5D, SERTAD2, ADRA1B, RNF149, HLA-DQA1, C2orf68, WIPI2, PLA2G3, MDGA1, C5orf49, TCAF1, ARHGEF15, HFE, ABHD14A-ACY1, CHRNA6, DOCK11, SLC36A1, USP17L2, STAMBPL1, KANK2, C1QTNF7, CAMK2B, SNX33, TSPAN17, PTX3, FAM216A, TMPRSS13, CHSY3, GRB2, MUL1, TEX15, HTR3A, RNF216, PIK3CD, MAN1C1, SUPV3L1, BCL2L15, CXCR3, CYFIP1, LARP4B, ZSCAN9, SLC38A11, SLC25A43, EPDR1, KMT5B, NAXE, TAF9, MVK, PNPLA1, ETNPPL, NAP1L3, CYP2E1, TAB2, MX1, PILRB, PRND, SIGLEC5, PLEKHA6, TPM2, NEB, PSPC1, ARHGAP9, PELO, TNFRSF4, LGALS3BP, SPRR2A, CARD17, HNRNPH1, PYGL, ANXA11, USF1, TPM1, LENEP, SEC31B, NVL, APLF, EFNA5, LPCAT2, SLC37A2, IFIT3, KRT23, TAPBP, CAPN1, ZNF730, TFIP11, CHST4, GATAD2A, FXYD1, TRIM16, DDX60L, RCOR2, PLCXD1, NDUFA7, CDC42BPB, H1-7, RNF39, TRIP12, PAX8, HIPK3, SCEL, STIMATE, ASL, LIN7B, C6orf163, SMOX, MMP1, SPIN3, RBM6, CCDC102B, HAS2, TBC1D26, ACADSB, IFT22, ARHGAP42, PAX1, CPA5, CPT1B, SULT4A1, GPX3, MYO1C, SPRR1A, C2orf76, TPH1, FLT4, IL1A, CCNA1, HIPK2, RHOV, PTGES, LIMK2, MBD2, MUSTN1, NTM, ZNF684, COPS5, MB21D2, DERL1, DNAAF5, AP3B2, SNX29, HRH3, IFNAR1, EXOC4, TESPA1, PMS1, C2orf92, KNTC1, MVP, HLA-DRB1, ACTR3, NR3C1, SERINC2, MYT1, CKAP4, BSN, FCRL6, MUC21, FAM221A, CD28, COPS8, IFI30, DACH2, WNT7A, EMCN, RNF130, TLE1, SCAMP2, LTB, CD274, TIGIT, CRYBG2, PTS, CXCL9, AQP12A, GALNT12, ULBP2, TMEM69, ADA2, DRICH1, HDAC9, ZNF852, SMURF2, ARHGAP29, AKAP7, DCAF4L1, WNT8B, HRH1, TTYH1, DCAF16, GLMN, FLT3, DAP, SPATA25, ATP6V1G2, PROS1, AVEN, DTX3L, RAB5C, GADD45B, BATF2, WDR44, PPP1R16B, NEGR1, SIRT4, MMP3, DUSP19, PSD4, CCDC190, MAP3K21, LILRB1, IFITM10, SLC8A1, SPART, DUSP7, ATP6V1B1, ILK, METTL8, P2RY2, TET1, C11orf42, PFKFB2, GLUD1, C2orf50, CD81, TARBP1, COL25A1, TRIM48, TM6SF1, GPC2, TEX9, KRTAP5-1, TEKT2, NUP50, MPEG1, DAAM1, TOGARAM2, PIP5K1A, CHN1, ZNF853, TMEM155, YAP1, VSNL1, SMIM8, BGLAP, MGAT4D, FCRLB, HSPA12A, TIAM2, BCO1, CASKIN1, JOSD1, IMPA2, RELCH, NRM, THEMIS2, SULT1B1, ABCC3, ATP8B4, HMBOX1, PGF, ACVR2B, FGR, CYSLTR2, WASL, ZCCHC3, SPRR2D, CATSPER1, CXXC1, NPIPA7, HVCN1, MMP23B, EPHB4, GALNT2, RPRD1A, QSOX2, TLR6, PAK2, NAA16, APP, C10orf67, BTK, DCLK3, APOE, TMEM30B, MAN2B1, PCDHGB6, RNFT2, PTP4A3, PSMB10, SCD, TCP11L1, HSDL1, SUSD3, PJA1, TAS1R1, SLFNL1, MX2, MEIS1, RALGPS1, DNAH14, RETNLB, MYO5B, DENND2D, TP53TG5, ZPBP, ASPH, CDCP1, MDN1, TLCD3B, GBA2, MAP3K20, POGLUT2, NEFM, SPRED1, RTCB, SLC44A1, PLPP5, S1PR3, TBC1D3C, OGDHL, PPP1R9B, FBXL22, YWHAG, BTBD17, FTH1, TSPAN14, XPR1, DCAF8, TWSG1, ACSF2, CD3D, JAML, THBD, MFSD4A, CPQ, FAM25A, GHRL, KCNC1, ZNF778, CLEC2L, ZNF443, FBXO46, CXCR4, CETP, SEZ6, GADD45G, AQP12B, ZDHHC23, RGS20, BCL2L11, MUTYH, DNER, OAS3, THUMPD2, USP49, CD2, SH2D3A, RFLNA, MYL12A, RAB40AL, TOB2, ZNF596, CFL1, C3orf80, TMIGD3, MEIOC, SERPINA5, SMURF1, LRFN2, BSND, ABCA13, TCERG1, PDE5A, SYNJ2, PLA2G12A, CLEC4C, HMGB3, ANKK1, BRIX1, FANK1, SPAG1, CMC1, FHL1, HHLA2, CAPN13, ZNF267, MAP1S, STK39, PPP1R32, KLF4, CYTIP, ICAM2, NEBL, HRH2, EFCAB12, TFPI, KIF26B, ACSBG1, ATP11B, BACH2, SESN2, ZNF77, PKM, SPRR3, ACOXL, GGACT, SLC1A2, YIPF1, PKD2L2, SLC43A2, KLF17, NEMP2, AP1S3, EXOSC2, SIGLEC1, CFHR4, BHMG1, JADE2, HPSE, STK38L, C5orf63, TMEM123, EXD1, PSTPIP1, PCMTD2, YKT6, PRSS22, SNCG, C3, CST4, EFCAB8, LLGL1, HSD17B2, BBX, ATXN10, S100A4, CRHR2, STKLD1, SLC25A4, CDX1, PRSS21, GRAMD2B, ZMAT3, EXTL1, PRR32, CDR2L, TRIP11, DRC3, XPNPEP2, FSIP2, GPR161, CHPF2, CBLN4, LRG1, TMEM144, SAT2, APOA2, SAA4, IRF1, MAPK3, SH2B2, NPRL2, NMBR, LIN28B, GPSM3, CPA2, CD226, ATOX1, APOBEC3D, PTPRU, HMCN1, INPPL1, CXorf21, MINDY2, KCTD19, HPS5, SLC45A3, PCNX1, RAF1, KLF7, TRPA1, PTPRCAP, H2BC4, ARHGAP27, TRPS1, STS, LIPK, PABPC1L2B, AAMDC, NRROS, RBBP6, LURAP1L, TMOD3, EXOSC3, CALCOCO2, SPATA17, SYNE3, OSBPL5, DCLRE1B, BTBD2, SCFD2, PALM2AKAP2, PCSK1, CDC42EP3, PTPRD, GRID1, SLC25A14, MEP1B, ZCCHC8, GNGT2, CRYBA2, RPS6KL1, TEDDM1, CYP26B1, CD247, TAC4, DYDC2, ADA, SEPSECS, AC011498.4, HAPLN2, RUFY3, RIMS3, ZGRF1, UBALD2, ZNF493, IFNA5, NPIPB13, FOXJ1, FGF21, CEND1, IQCB1, NEDD4L, SLC1A1, TNFRSF8, STOM, IQCM, TXLNB, EVA1C, PDE6C, SLC2A1, PNMA8C, EGFLAM, WDFY4, FAM207A, POU3F1, LANCL2, P2RY10, MAPK1, KLRF2, GLI3, KCNH2, RPS21, AP2A1, KLF2, CYRIA, NOP56, TAF1D, PON2, TLR2, C4B, GALNT18, PEAR1, SOX12, FOXF2, SETDB1, ORAI3, KCTD10, LUC7L3, B3GNT9, CREG2, CCDC130, C4A, CNOT4, NCAM2, SLC12A9, LGMN, EFCAB11, NDEL1, PPOX, NFIC, RAMP1, CTNS, TMEM151B, PDHX, F2, CELSR1, CCL13, CXCL16, ETV1, C10orf95, CLIC1, TFB2M, POLG2, DDX59, LEUTX, ZBED5, TUBD1, FP565260.3, CCL5, SLC8B1, IL36G, STRBP, CNTNAP3B, DENND2B, TRNT1, YJEFN3, PCDH17, AGAP2, PBK, SPOCK2, SMAP2, RB1, PKP2, ATP12A, IFITM5, ZAR1L, ZNF322, NDUFAF7, SRSF12, SST, TCIM, POC5, EEF2KMT, URGCP, FUT3, MYORG, ZFYVE26, COX14, PLEKHM2, CD300LB, GLRA3, ELOA3B, HSD17B1, SELENON, SCART1, CYTH1, TTPA, SLC5A2, GTPBP3, C19orf33, SYNE4, SFXN2, AGPS, PFDN2, TBC1D5, ADAMTS15, PIK3R1, C2orf15, MBNL3, SH2D1A, SFXN4, ISY1-RAB43, UFSP2, SERPINB1, CNGB1, GDF15, COL4A6, CETN2, ZCCHC10, ACOT12, ARHGEF5, UBAC2, ATL3, PRUNE1, PAIP2B, DENND4B, DOK3, SOWAHB, PORCN, NPC1L1, FOXJ2, SLC2A14, FAM186A, UQCRB, COPS7A, WDR17, LIMD1, SLITRK6, FBN3, FREM1, CD52, CXXC5, GTPBP4, ZNF575, EVC, SERINC3, GOLGA6L10, ARHGAP28, ZNF669, SLC4A9, SRL, MRPS25, FRMPD1, CPNE8, MICAL2, SHISA5, LIN9, ADRB3, NKTR, PROX1, CREBZF, B3GALNT1, AKNA, CTNND2, CELF4, COBL, RWDD2A, ANKRD44, HLA-DMA, SMAD1, SPRED3, FAM25C, TTLL10, APEX2, PHKG2, CD48, CTBS, BBOX1, RAB39B, PRLHR, ST18, IFI27L2, LRFN3, ARL14, TLNRD1, NACAD, C2orf74, TNFSF9, ELAVL3, PLAC8L1, IRX5, RAMP3, TMEM184C, CSRNP3, ATP13A2, SUGP2, RFC5, STK24, TSPAN6, TICAM1, IL37, ZC3H7B, GSTA4, SPTY2D1OS, PI16, DNAJB6, NOX3, SERPINB7, NAE1, TRIM4, GCC1, FES, RASSF4, PABPC1L2A, ATP7A, MDH1B, TIMD4, B4GALT1, SPAG8, HMCN2, SLC35F5, FCN1, CA6, RPP14, TMEM167B, CTNNBIP1, ANXA2R, CKS2, ZNF764, TEN1-CDK3, SLC7A2, SH3RF3, NEIL1, CD300A, ANKRD49, EPN1, RNF182, FBXO6, SLC16A4, ARHGDIA, CLEC12A, MPZL1, MAT2B, PIF1, GNPTG, KCNIP1, COX6C, AMER3, ZNF385A, HGFAC, CHST12, DGKA, APOH, HGF, MIEN1, IL1RAP, TRAPPC2, KLRB1, DNAH17, NEDD4, ANGEL2, PGBD4, ASXL1, EFNA1, MOCOS, PIK3C2B, SRSF7, SLC9A3R1, CEP19, CYP2U1, STAC3, TMEM35B, DEGS1, PAQR6, BRI3, METTL7B, PSMC2, CREB3L2, NUP88, NEUROG3, SOX14, SYT4, PPP2R5E, COX6B2, SLC19A2, NR2C1, ERICH6B, IKBKE, PDP2, SPTBN1, SERPINA3, LAMP1, C1GALT1C1L, PNMA8B, SEPTIN6, INIP, SGK1, RAB19, ITK, VPS18, FOXC1, CEP70, DIPK1C, CIITA, B3GNT2, EFR3A, ZNF519, TOX3, SERPINB4, HPS6, AC005726.1, ASMTL, IL33, PDHB, CLCN4, DCUN1D3, RSPH14, C4orf3, RGS9, RTBDN, KRT85, TBC1D10A, ZNF483, ZNF711, ACO2, KLRC3, FLVCR2, GPR135, NXF1, FANCG, RNASEL, CFAP300, MMP26, LARGE2, SORBS3, ARNT2, SP140, PDLIM1, WT1, CREBL2, DLEC1, PAQR7, PCSK5, HIC2, ARL16, SCRN1, EGF, FOXQ1, ARGLU1, IL22RA1, LOXL3, FAM78B, AREG, C7orf26, SPATA1, ZFYVE27, ACE, MGAT1, RIBC2, HPDL, SOAT1, APCDD1L, SLC6A14, RNF5, SIT1, LRRC69, CBWD6, PLPPR5, FERMT2, AP002495.1, PHF8, AC010542.3, SLC25A3, MYLIP, TNNI2, EPPIN, BTAF1, FBXL13, OR10G2, TRADD, LIN28A, SOD3, SNX3, PFN1, MTARC1, HNF1B, DNAAF1, SLC38A2, AGAP9, CAPN12, FAM76B, MAPKAP1, MTUS1, ARHGAP19-SLIT1, CCPG1, PEX11G, FCMR, EFCAB6, MACC1, CEBPB, PAX7, ATXN1, AL355987.3, RHBDD1, ADAMTS4, HECTD4, TMEM272, AP5B1, DMTF1, CCDC85B, IFI44L, PLXNB1, CREB5, FKBP7, MAST1, FUOM, EZR, CCRL2, GPR82, STK38, OAS1, CYP39A1, CFAP97D1, TLL1, ADGRG1, POPDC2, SQLE, FAM102B, AC068775.1, AC023490.5, TMEM158, LY6K, PFAS, DCTN6, S100A12, UNC80, SERINC1, TPH2, FRRS1, SLC2A5, DEPTOR, STXBP1, NSG2, ZBTB3, FAM20A, NECTIN1, KRT28, TMEM236, MYBPC2, RILPL2, MESD, AMZ1, LHFPL3, TPD52, PMPCA, IL1RL1, SIRPB1, P3H3, SH3GL2, SLAMF7, YIPF5, DEPDC4, INTS10, LRIT3, LILRA2, PDE1B, MACIR, CFD, CLCN3, AATF, COL13A1, CEP95, FABP12, ADD1, KLF13, IL17C, NCF1, GLDC, DAGLB, TMEM174, PTTG2, GPR148, CD27, C15orf39, ZNF700, TRIM67, MARCHF10, DNAJC3, NDUFS3, MBOAT4, ANKIB1, RFTN2, ZNF121, INHBB, ZNF732, ADAMTS14, TMEM176B, NUB1, FTSJ1, LTBP4, MCMDC2, AOX1, COA8, AC068831.6, PKLR, ZNF709, DPM2, RAD17, LIPG, BCL2L14, HEYL, ABT1, LHX6, CLSTN2, LSAMP, RAD52, TIMM21, MTG2, VAMP3, THRA, C15orf48, ARHGEF25, ARHGAP17, TEX101, MS4A2, IQCK, HSPB1, EPS8L1, GLTPD2, RPS6, STYXL1, CD164L2, LILRA6, ARID5B, VPS13A, TEP1, NKX2-2, PLOD3, MRPS21, TPRG1, KCNQ2, SESTD1, PCSK6, CEP112, PARP11, KLF6, ANO10, CCR3, ADH1C, ADGRE1, HLA-DQB1, GRK3, NR5A2, CTSZ, AC027796.3, RNF185, RALGAPA1, TTC17, CD3G, TIAL1, KCNK9, TNIP3, FRG2B, TRIM49D1, UGT3A2, MAP3K3, UGT1A6, UBTD2, RETSAT, SLC26A5, ARFIP2, PLEKHG3, OGFOD1, POGZ, TIMM17A, AIM2, ZNF705G, SRR, STYX, KRTAP5-9, RAB15, BARX2, PLEKHD1, CNN3, DPEP2, BORCS8-MEF2B, LMBR1, ZNF365, OSTF1, CALY, PSME2, TBC1D24, INSIG2, STOML2, TAF11, MUC20, CD5, ADAP1, EPHA10, ITM2A, COX17, CPNE6, ZNF662, MSANTD2, SHROOM2, FKBP9, NNAT, ZNF76, LEPR, ZDHHC7, RAB44, ANKRD33, KRTAP13-4, BIVM-ERCC5, PLA2G4F, HOXB4, CXCL13, SIRPG, UBASH3A, CDHR3, ITGB7, IFT81, ZKSCAN8, ZNF484, GPR142, PANK1, COQ8A, RANGAP1, DDX39B, CBR3, FOXF1, TMEM256-PLSCR3, CLCN6, PAQR4, APOBEC1, RAP1GAP2, SLC47A2, ZFYVE21, WDR88, ZNF682, TRAT1, SLC16A7, TM4SF1, FRAT2, SLC5A11, LCK, ZNF699, P3H1, TMPRSS4, APOC4-APOC2, ANAPC4, NLRC4, LYZL2, POLD4, MYL4, PLCL2, BRICD5, KRTAP10-4, PHYHIP, CCND3, ADAM15, TRMT13, TBX4, SLC35A1, ORC6, SNX18, EFR3B, CDH6, ZNF560, ASPSCR1, STON1, MMP24, PDCD1, PHTF1, OASL, ESYT3, ANKRD2, ZNF714, PPM1M, STUB1, ZNF534, AC026464.1, GAS2, F2RL1, SLCO5A1, CD47, MYO15A, PARPBP, CASP5, MMP19, PPARA, VAV3, ZNF24, AP1S2, DTX4, HK3, ZNF284, C8orf88, SCD5, ZBED2, DHPS, SPTLC2, RALBP1, CPEB1, RRNAD1, BDH1, OSR1, SPATA5L1, IFNW1, NOL4, GPR37, SLC12A3, UIMC1, EIF6, LHX5, ZP1, ZHX3, PTGFRN, CRYAA, TOGARAM1, PLD1, SLC38A8, NR2F1, PP2D1, KIF1C, FOXL1, C19orf73, CBWD3, MYMK, MAGEA3, JAGN1, METTL4, MORC3, SACM1L, PRAM1, ABI1, GRINA, DPYSL5, CUZD1, ACTR1A, IL17RB, FRMD1, USP5, CCL23, MEX3C, ASB16, SHANK3, SMAGP, MB, TRIM69, SEL1L, NDUFA2, PALM, SYS1, HEMGN, ACADVL, GBP5, CMIP, BMERB1, KLK15, TCTEX1D4, HS3ST1, TMEM97, SDCBP, IQSEC2, PPP3CA, FGD4, TRIM71, TRAF7, NHLRC2, NT5DC4, C9orf131, R3HDM4, CCNJ, SIAH3, CLDN12, H3-3B, SLC35C1, DOK1, MITD1, SSR2, PLAGL1, ADAT2, RSPH6A, AK6, SRD5A2, PRR5, KRTAP5-5, SNX24, CELA3A, SLC27A1, FLT1, C1orf216, CDKN1C, RRP7A, RTN4R, CLEC9A, HDAC7, ETHE1, RTP4, TRIT1, PLP2, ATAD3B, METRN, CCDC114, SULT1E1, TERB1, INSL6, GPR78, CELF2, HHIPL2, OXGR1, DSC1, TMEM196, HSPA14, MTNR1A, RFX6, TTC7B, CARD10, TAS2R14, RARB, H2AW, FGF14, CORO7, CD244, IL1R2, DOK6, TPBGL, ACTR6, TUBB, CNIH1, ARRDC2, LRRC3C, XYLT1, GEMIN2, KCNAB3, C20orf203, RASGRP3, NIBAN2, PMF1-BGLAP, TOR4A, CMTM6, PTRHD1, PANX1, DNAJC5B, RBM5, TP53AIP1, TADA1, RPS24, RPL7A, MAK16, TMEM184B, ARHGEF40, TRIB1, NAT1, CAP2, RBMX, CCDC148, RBP5, FNDC3B, AREL1, GID8, GMCL2, COL21A1, VWA3A, SEC62, AQP10, ALG5, CD58, FAM32A, HSPE1, IQSEC1, SGIP1, SGSM1, KPNA1, ARSA, RFX4, SEC31A, NUF2, FOXN3, USP38, CLCNKA, CD72, FBXO31, FUT6, GOLGA6L4, CA14, GPRC5A, UBE2E2, FAM126A, COP1, TMED6, NPIPB12, SHROOM4, LIPA, EFHC2, ARHGEF18, TDP1, CD1C, ZNF84, KCNK4, YY1, RAB36, ATP1A2, LRP2BP, LPO, COX6A1, DHFR, TLR5, AKT2, EPS8, GSE1, KCNB2, CEP170B, UBQLN2, RNF133, CLEC3A, PODXL, ZNF683, RAX, CRYGB, ZNF337, SRSF10, C19orf25, TAX1BP1, ARHGEF4, AP1S1, E2F1, RIC3, PPIC, CRYBG3, ARHGAP5, CEP41, SIX2, TM4SF5, CIART, IMMP2L, RCN3, DNAL4, EVX2, PRKAR1B, COLEC11, ANKRD33B, GBGT1, CCP110, APOC3, SGCB, LRRC75A, IGF2BP2, PDE4B, SLMAP, PPP1R3C, NEUROD1, SLC35G1, WIPI1, ACSM2A, STARD3, LTF, TNPO3, SPIB, CERS6, CTAG1B, ING5, RNF112, SRGAP2, HOOK1, RXRA, LRPPRC, MAOB, TMEM265, LMO7, UGP2, APBA1, CD6, AKT3, TOLLIP, CEP152, SETD6, RAPGEF1, SEC24D, KANTR, MS4A7, FUCA1, MAPK12, MRAS, TAF11L12, LRRC66, ACTG1, DIO3, PIGBOS1, AC036214.3, EMC9, MIOS, NF2, TMEM231, DUSP28, OAT, B4GALT3, CHST6, VPS9D1, MZF1, GOSR2, IQSEC3, NEURL3, CATSPER2, TRAF3IP3, DSC3, AL162231.1, GFM2, IFI44, TMEM43, GBP7, RPL14, DNAJA4, SERPINB10, LRRC10B, GATA1, YWHAB, TRIM56, SOX3, RALGPS2, RRAS2, SRP68, PTEN, DIAPH1, DENND2C, ANGPTL5, ABCA8, NCR3LG1, FRG2, ZC3H11B, NUDT18, GLI2, RPL5, BORCS6, CIAO2B, F5, CHRD, JMJD7-PLA2G4B, BLOC1S5-TXNDC5, CCDC134, EEF1E1, TSPAN4, PIGV, C2orf42, INS-IGF2, ISG15, KIRREL3, SPOCD1, CFP, RAET1G, KAAG1, AC004832.3, TNFAIP2, IL9R, SLC7A11, SLC50A1, ARHGAP33, SLC46A3, PTK2B, PFN4, MYO1G, CTXN2, PROSER1, ANKHD1, LEF1, LRRC7, GIPC1, EIF2B2, EPB41L4A, GIN1, RPS7, LENG9, RFX8, SFPQ, CTSL, FMC1, UQCC3, AC008764.4, PRDM11, CRBN, ERICH6, CARM1, NDP, MTMR6, GRHL3, FAM160A2, TMEM88B, S1PR4, LIPT2, VWA5B2, ZDHHC4, ZKSCAN3, SERPINB8, CDPF1, SLC15A4, TUBB3, TMIE, FGF17, SLC4 |
